# The therapeutic potential of natural metabolites in targeting endocrine-independent HER-2-negative breast cancer

**DOI:** 10.3389/fphar.2024.1349242

**Published:** 2024-03-04

**Authors:** Mirosława Püsküllüoğlu, Izabela Michalak

**Affiliations:** ^1^ Department of Clinical Oncology, Maria Skłodowska-Curie National Research Institute of Oncology, Kraków, Poland; ^2^ Wrocław University of Science and Technology, Faculty of Chemistry, Department of Advanced Material Technologies, Wrocław, Poland

**Keywords:** HER-2 negative breast cancer, anticancer drugs, natural metabolites, chemotherapy, targeted therapy, nanomedicine

## Abstract

Breast cancer (BC) is a heterogenous disease, with prognosis and treatment options depending on Estrogen, Progesterone receptor, and Human Epidermal Growth Factor Receptor-2 (HER-2) status. HER-2 negative, endocrine-independent BC presents a significant clinical challenge with limited treatment options. To date, promising strategies like immune checkpoint inhibitors have not yielded breakthroughs in patient prognosis. Despite being considered archaic, agents derived from natural sources, mainly plants, remain backbone of current treatment. In this context, we critically analyze novel naturally-derived drug candidates, elucidate their intricate mechanisms of action, and evaluate their pre-clinical *in vitro* and *in vivo* activity in endocrine-independent HER-2 negative BC. Since pre-clinical research success often does not directly correlate with drug approval, we focus on ongoing clinical trials to uncover current trends. Finally, we demonstrate the potential of combining cutting-edge technologies, such as antibody-drug conjugates or nanomedicine, with naturally-derived agents, offering new opportunities that utilize both traditional cytotoxic agents and new metabolites.

## 1 Introduction

Breast cancer (BC) in females has emerged as the most frequently diagnosed cancer, comprising almost 12% of all new cases. Among women alone, BC resulted in nearly 650,000 deaths globally in 2020 (Caswell-Jin et al., 2018; Sung et al., 2021). BC subtypes differ in terms of prognosis and treatment strategies. Depending on the expression of the estrogen receptor (ER), progesterone receptor (PR), Ki67 antigen, and Human epidermal growth factor receptor-2 (HER-2), BC can be divided into Luminal A&B (70% for HER-2 negative cases), HER-2 positive (around 15% including ER/PR + HER-2+ BC), and triple negative (TNBC) (10%–15%). Around 30% of BC patients experience metastatic disease during follow up (20%–30%) or *de novo* (5%–10%). BC mortality rates vary by subtype, with TNBC at around 30%, luminal B at 20%–25%, HER-2-positive at 20%, and luminal A at 10%–15% (Hennigs et al., 2016; André et al., 2019; Gnant et al., 2022; Wynn and Tang, 2022; Hassan and Ates-Alagoz, 2023). Patients with TNBC have the shortest survival. In contrary, HER-2 negative Luminal BC carries a good prognosis. Patients with Luminal BC, who have developed resistance to hormonal agents face an unfavorable situation and the course of such endocrine-resistant BC is more like TNBC (André et al., 2019; Hassan and Ates-Alagoz, 2023). This cluster of endocrine-independent HER-2 negative BCs represents an unresolved clinical challenge, characterized by a dearth of treatment alternatives based on chemotherapy agents (Cardoso et al., 2020; Cortes et al., 2020; Modi et al., 2020; Bardia et al., 2021; Corti et al., 2021; Michalak and Püsküllüoğlu, 2022; Püsküllüoğlu and Michalak, 2022; Siddiqui et al., 2022). Majority of currently used drugs serving as a backbone to the most effective approved options are naturally derived (Table 1, based on product characteristics, accessed on 5th September 2023) (Cortes et al., 2020; Modi et al., 2020; Pullaiah and Raveendran, 2020; Rugo et al., 2020; Bardia et al., 2021; Corti et al., 2021; Ibrahim, 2021; Michalak and Püsküllüoğlu, 2022; Püsküllüoğlu and Michalak, 2022; Sekar et al., 2022; Siddiqui et al., 2022; Aloss and Hamar, 2023; Dogra and Kumar, 2023; Kciuk et al., 2023).

**TABLE 1:** Drugs derived from natural resources used in the treatment of TNBC and hormonal-resistant HER-2 negative BC.

Drug			Source		Current production method	Formula	Mechanism of action	Side effects	Indication - examples	Ref.
Group	Name	Tradename	Kingdom	Species						
Taxanes	Paclitaxel	Taxol	Plant (shrubs and trees) and fungi	*Taxus brevifolia* Nutt., Taxaceae (bark), endophytic fungi: *Taxomyces andreanae* Strobel, A. Stierle, D. Stierle & W.M. Hess 1993, Meruliaceae, *Pestalotiopsis microspora*, Pestalotiopsidaceae, *Tubercularia* sp., Nectriaceae, *Phyllosticta citricarpa* (McAlpine) Aa 1973, Phyllostictaceae	Semi-synthesis	C_47_H_51_NO_14_	Prevents microtubule depolymerization leading to cell cycle arrest	Allergic reaction	Breast cancer	Dogra and Kumar (2023)
								Bone marrow suppression	Ovarian cancer	
								Peripheral neurotoxicity	Esophageal and gastric cancer	
								Liver toxicity	Pancreatic cancer	
									Head and neck cancer	
									Lung cancer	
	nanoparticle albumin-bound paclitaxel	Abraxane	Nanoform of paclitaxel Albumin bound paclitaxel					Neutropenia	Breast cancer	
								Peripheral neuropathy	Pancreatic cancer	
								Arthralgia/myalgia		
								Gastrointestinal disorders		
	Docetaxel	Taxotere	Plant (shrubs and trees)	*Taxus baccata* L., Taxaceae	Chemical synthesis	C_43_H_53_NO_14_		Alopecia	Breast cancer	
								Liver toxicity	Prostate cancer	
								Bone marrow suppression	Lung cancer	
								Peripheral neurotoxicity	Stomach cancer	
								Fluid retention		
Vinca alcaloids	Vinorelbine tartrate	Navelbine	Plant (flowering plant)	*Catharanthus roseus* (L.) G. Don, Apocynaceae	Semi-synthesis	C_45_H_54_N_4_O_8_	Prevents microtubule polymerization	Bone marrow toxicity	Breast cancer	Dogra and Kumar (2023)
								Peripheral sensory neuropathy	Lung cancer	
								Alopecia	Lymphomas	
								Gastrointestinal disorders		
Camptothecin analogues	Irinotecan (CPT-11)	Camptosar	Plant (shrubs and trees)	*Camptotheca acuminata* Decne., Nyssaceae	Semi-synthesis	C_33_H_38_N_4_O_6_	Topoisomerase I inhibitor	Gastrointestinal toxicity including diarrhoea	Colorectal cancer	Modi et al. (2020), Pullaiah and Raveendran (2020), Rugo et al. (2020)
								Bone marrow suppression	Pancreatic cancer	
									Gastric cancer	
	SN-38	^a^Trodelvy	As above		Synthetic analogue of CPT^1^	C_22_H_20_N_2_O_5_		^a^Neutropenia	Breast cancer	
								Diarrhea		
								Nausea and vomiting	Urothelial cancer	
								Allergic reactions		
	Deruxtecan	**Enhertu				C_52_H_56_FN_9_O_13_		**Lung interstitial disease/pneumonitis	Breast cancer	
								Neutropenia	Gastric cancer	
								LVEF decrease		
Anthracyclines	Doxorubicin hydrochloride	Adriamycin	Bacterium	*Streptomyces peucetius* ssp. *caesius*, Streptomycetaceae	Semi-synthesis	C_27_H_29_NO_11_	Topoisomerase	Cardiac toxicity	Breast cancer	Aloss and Hamar (2023), Kciuk et al. (2023)
								Bone marrow suppression	Leukemias	
								Alopecia	Lymphomas	
								Nausea and vomiting	Sarcomas	
	Liposomal doxorubicin hudrochloride	Myocet	Liposomal form of doxorubicin			Liposomal doxorubicin	II inhibition	Bone marrow suppression	Breast cancer	
								Mucositis	Ovarian cancer	
								Cardiac toxicity	Kaposi’s sarcoma	
	Epirubicin hydrochloride	Ellence	Bacterium	*Streptomyces peucetius*, Streptomycetaceae	Chemical semi-synthesis from daunorubicin	C_27_H_29_NO_11_	Generation of free radicals	Bone marrow suppression	Breast cancer	
								Mucositis	Lymphomas	
								Alopecia	Sarcomas	
								Cardiac toxicity		
Podophyllotoxin derivative	Etoposide	Vepesid	Plant (flowering plants)	*Podophyllum peltatum* L., Berberidaceae (root)	Semi-synthesis	C_29_H_32_O_13_	Topoisomerase II inhibitor	Myelosuppression	Lung cancer	Dogra and Kumar (2023)
								Nausea and vomiting	Testicular cancer	
								Alopecia	Leukemias	
								Secondary neoplasms	Lymphomas	
									Neuroblastoma	
Polyketides Epothilone derivative	Ixabepilone	Ixempra	Bacterium (Myco-bacterium)	*Sorangium cellulosum*, Polyangiaceae	Semi-synthesis	C_27_H_42_N2O_5S_	Prevents microtubule depolymerization leading to cell cycle arrest	Peripheral sensory neuropathy	Breast cancer	Ibrahim (2021)
								Fatigue		
								Asthenia/myasthenia		
								Alopecia		
								Nausea and vomiting		
								Hand-foot syndrome		
								Myelosupression		
Macrocyclic ketone analogue	Eribulin mesylate	Halaven	Animal (marine sponge)	*Halichondria okadai* Kadota, 1922, Halichondriidae	Synthetic analogue of halichondrin B	C_41_H_63_NO_14_S	Prevents microtubule polymerization	Bone marrow suppression	Breast cancer	Sekar et al. (2022)
								Peripheral neuropathy	Liposarcoma	
								Gastrointestinal toxicity		
								Mucositis		

^a^ data for sacituzumab govitecan-hziy, ** data for Fam-trastuzumab deruxtecan-nxki Abbreviations: 1CPT, camptothecin.

^b^ LVEF, left ventricular ejection fraction.

Medications such as check point inhibitors or targeted drugs have not yielded the anticipated breakthrough in therapy, while newly registered antibody-drug conjugates (ADC) still rely on chemotherapy agents derived from living organisms (Cortes et al., 2020; Modi et al., 2020; Pullaiah and Raveendran, 2020; Rugo et al., 2020; Bardia et al., 2021; Corti et al., 2021; Ibrahim, 2021; Michalak and Püsküllüoğlu, 2022; Püsküllüoğlu and Michalak, 2022; Sekar et al., 2022; Siddiqui et al., 2022; Aloss and Hamar, 2023; Dogra and Kumar, 2023; Kciuk et al., 2023).

**FIGURE 1:**
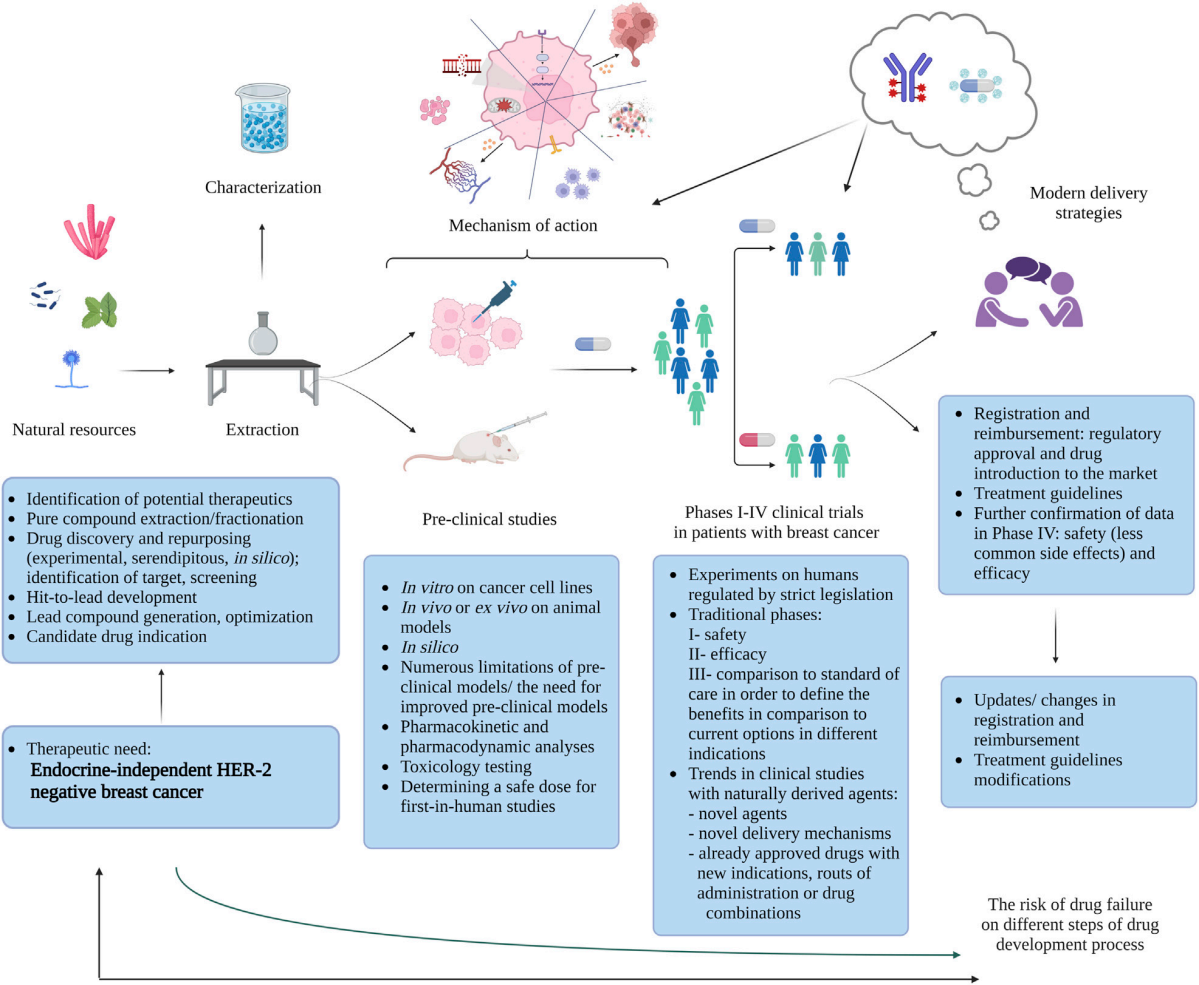
Bench-to-bedside journey: natural metabolites for endocrine-independent HER-2 negative breast cancer treatment (Berdigaliyev and Aljofan, 2020; Choudhari et al., 2020; Kaushik et al., 2021) (created with BioRender).

In the recent years, there has been a significant advancement in the development of novel natural-derived drug candidates aimed at tackling the unmet challenges in treating TNBC and HER-2 negative, endocrine-resistant BC. The pathway from metabolite identification to drug registration (Figure 1) is a multistep and long process. Majority of metabolites showing potential in pre-clinical setting will fail to meet endpoints in clinical trials. In this review we aim to show that the recent development in this field can be attributed to each step of this translational process: from bench to bedside (Berdigaliyev and Aljofan, 2020; Choudhari et al., 2020; Kaushik et al., 2021). We present natural resources that can be a source of biologically active metabolites and the significant pre-clinical activities exhibited by these metabolites, offering insights into their intricate mechanisms suggested in recently published studies. Moreover, we identify trends in ongoing clinical trials that incorporate naturally-derived drugs. We also shed light on the potential role of cutting-edge technologies like antibody-drug conjugates or nanomedicine in diminishing the toxicity and improving efficacy of old and newly discovered naturally-derived agents making predictions about future directions.

## 2 The current paradigms in the treatment

### 2.1 General treatment guidelines

Systemic treatment in TNBC radical setting is recommended for tumors larger than 5 mm or node-positive disease. Preferred regimens involve anthracycline-based and taxane-based therapies, capecitabine and, in some cases, novel agents in the (neo)adjuvant setting (NCCN Clinical Practice Guidelines in Oncology, 2023; Triple-negative Breast Cancer, 2023).

### 2.2 Single agent schemes

In patients with metastatic endocrine-independent HER-2-negative BC with minor cancer-related symptoms and with a limited tumor burden, sequentially used single-agent schemes are preferred (Cardoso et al., 2020; NCCN Clinical Practice Guidelines in Oncology, 2023). The order of agents is not established and depends on numerous factors, for example, previous treatment received in adjuvant and metastatic setting, the risk of cross-resistance, *Breast Cancer gene 1/2* (*BRCA1/2*) mutation status, or comorbidities. Typical options include (Cardoso et al., 2020; NCCN Clinical Practice Guidelines in Oncology, 2023; ER-positive HER2-negative, Breast Cancer, 2023; Triple-negative Breast Cancer, 2023):

• Taxanes: docetaxel, paclitaxel or nanoparticle albumin-bound (nab)paclitaxel.
• Anthracyclines: doxorubicin hydrochloride (doxorubicin) or its pegylated, liposomal form; epirubicin hydrochloride (epirubicin).
• Capecitabine (an oral prodrug of fluorouracil).
• Vinca alkaloids: vinorelbine tartrate (vinorelbine) as intravenous infusion or oral capsules.
• Gemcitabine hydrochloride (gemcitabine).
• Eribulin mesylate.
• Etoposide as oral form.
• Platinum agents: cisplatin, carboplatin.
• Ixabepilone, not available in Europe.

Other agents used include:

• The Poly (ADP-ribose) polymerase (PARP) inhibitor olaparib in *BRCA1/2* mutated patients.
• ADC Fam-trastuzumab deruxtecan-nxki (T-DXd) for pretreated patients with HER-2 low status (1+ or 2+ in immunohistochemistry and *in situ* hybridization negative status) as per the Destiny-Breast04 study in ER/PR-positive and ER/PR-negative BC (Modi et al., 2020).
• ADC sacituzumab govitecan-hziy (SG) for metastatic and pretreated HER-2 negative ER/PR-positive BC as per the TROPiCS-02 study (Rugo et al., 2020) and for TNBC as per the ASCENT trial (Bardia et al., 2021).
• Pembrolizumab, anti-Programmed cell death protein 1 (PD1) monoclonal antibody (mAb) as per the KEYNOTE 355 study in TNBC in combination with chemotherapy (Cortes et al., 2020).

### 2.3 Combination regiments

For patients who require combination regimens due to rapidly progressing disease, symptoms or large tumor burden, there are numerous drug combinations available (Cardoso et al., 2020; NCCN Clinical Practice Guidelines in Oncology, 2023; ER-positive HER2-negative, Breast Cancer, 2023; Triple-negative Breast Cancer, 2023):

• Anthracycline-based schemes (with taxanes, cyclophosphamide and fluorouracil).
• Taxane-based regimens (with gemcitabine, capecitabine).
• Platinum-based regimens (with taxanes, gemcitabine or vinorelbine).
• Ixabepilone with capecitabine.
• An old scheme known as CMF (cyclophosphamide, methotrexate and fluorouracil).

### 2.4 Naturally-derived agents

Numerous of the above-mentioned drugs have been derived from natural sources as secondary metabolites (Michalak and Püsküllüoğlu, 2022; Püsküllüoğlu and Michalak, 2022; Siddiqui et al., 2022). ADC such as T-DXd and SG, used in the discussed indication, can also be included in this group. The drugs conjugated with mAbs (trastuzumab directed against HER-2 for T-DXd and sacituzumab directed against Trophoblast cell-surface antigen 2 [TROP-2] for SG) are camptothecin derivatives (deruxtecan for T-DXd and SN-38 for SG) (Corti et al., 2021).

There is a long history of folk medicine with the usage of species being a source for anticancer naturally-derived agents. For example, the Himalayan yew (*Taxus wallichiana* Zucc., Taxaceae) bark and leaves are used in traditional medicine for steam baths (rheumatism), paste application (fractures, headaches), and medicinal hair oils. In Pakistan, stem decoction treats tuberculosis. Unani medicine uses them for Zarnab, addressing various conditions, while Ayurveda employs young shoots for a tincture treating headache, feeble pulse, giddiness, diarrhea, and severe biliousness (Juyal et al., 2014). Within the Ayurvedic medicinal system, various metabolites of periwinkle (*Catharanthus roseus* (L.) G. Don, Apocynaceae) are employed in traditional herbal medicine to address a range of health conditions, including diabetes, cancer, stomach disorders, liver, kidney and cardiovascular diseases (Kumar et al., 2022). The extract containing podophyllotoxin has been historically noted for its effectiveness as a laxative and as a treatment for diverse medical conditions, including tuberculosis, gonorrhea, menstrual disorders, dropsy, psoriasis, syphilis or venereal warts (Shah et al., 2021).

## 3 Secondary metabolites as anticancer agents

Research into natural metabolites with anticancer properties began in the 1960s and is still ongoing (Michalak and Püsküllüoğlu, 2022; Püsküllüoğlu and Michalak, 2022). Extracts obtained from plants, algae, fungi and lichens are a challenge because they are multi-component mixtures of active, partially active and inactive substances. Their composition may vary depending on the plant raw materials used; parts used (aerial parts, roots, bark, etc.); form of the biomass–fresh, dried, fermented; area, season, date, time of harvesting, etc., and the method of preparing these extracts (Heinrich et al., 2022) imitations can also include restricted availability of natural raw materials; structural complexity of secondary metabolites; stability concerns; purification, isolation and characterization difficulties; synthetic limitations or formulation hurdles. In order to ensure the reproducibility and interpretation of the results of pharmacological and clinical tests, Heinrich et al. (2022) proposed recommendations regarding the reporting of plant material and its method (stages) of processing.

### 3.1 The main groups of secondary metabolites

The main groups of secondary metabolites reported to have anticancer properties in endocrine-independent HER-2-negative breast cancer are phenolics, alkaloids, and terpenoids (Raman et al., 2018; Ding et al., 2020; Lee et al., 2020; Mazzei et al., 2020; Maungchanburi et al., 2021; Noh et al., 2021; Lin et al., 2022; Qi et al., 2022; Sancha et al., 2022; Youssef et al., 2022; Thilagavathi et al., 2023). Phenolics include simple phenols (phenolic acids and coumarins) and polyphenols (flavonoids and non-flavonoids such as tannins, lignans, and stilbenes). Examples of alkaloids, low-molecular-weight nitrogenous metabolites, are atropine, caffeine, capsaicin, cocaine, daturin, hiosciamin, lysergic acid, nicotine, strychnine, quinine (Alamgir, 2018; Thilagavathi et al., 2023). Terpenoids or terpenes, the major metabolites of essential oils, are classified as hemiterpenes (C5), monoterpenes (C10), sesquiterpenes (C15), diterpenes (C20), sesterterpenes (C25), triterpenes (C30), and tetraterpenes/carotenoids. This group of secondary metabolites includes aromatic oils, carotenoids, resins, steroids, waxes, and others (Alamgir, 2018; Thilagavathi et al., 2023).

### 3.2 Optimizing anticancer metabolite extraction

The key stage of research is the selection of biomass, which is a potential source of these anticancer metabolites. Subsequently, appropriate extraction techniques should be used to isolate metabolites of interest. In addition to simple extraction techniques, such as mixing/shaking, maceration or continuous extraction in the Soxhlet apparatus, novel extraction techniques, such as supercritical fluid extraction, sonication, ultrasound- and microwave-assisted extraction (Shin et al., 2017; Raman et al., 2018; Ding et al., 2020; Lee et al., 2020; Mazzei et al., 2020; Noh et al., 2021; Youssef et al., 2022) are in demand. The extraction process is usually carried out at room temperature in order to protect biologically active metabolites from degradation. Extracts derived from the biomass typically provide low yields of bioactive chemicals, which makes their isolation and purification time-consuming and expensive (Mazzei et al., 2020; Lin et al., 2022). Therefore, the selection of the appropriate extraction process, experimental conditions, and solvent seems to be of paramount importance. Finally, a detailed characterization of the obtained extract, which is a concentrate of biologically active metabolites, is required, or the isolation of metabolites from the extract showing desired anticancer properties specific to a given cancer type. The overriding goal is to search for extracts rich in bioactive substances that have an inhibitory effect on the proliferation and viability of cancer cells, while being comparatively safe for normal cells (Raman et al., 2018). Therefore, further fractionation of the extract is needed using the column chromatography separation. The purity of the active metabolites can be confirmed by a High-Performance Liquid Chromatography (HPLC). For the identification of different metabolites within an obtained fraction, gas chromatography coupled with mass analysis (GC-MS) can be applied. The functional groups in the isolated metabolites can be determined by Fourier Transform Infrared (FT-IR) Spectroscopy and the structure can be identified using Proton Nuclear Magnetic Resonance Spectroscopy (1H NMR) and other spectroscopic data (Maungchanburi et al., 2021; Heinrich et al., 2022; Qi et al., 2022; Sancha et al., 2022; Youssef et al., 2022).

The conducted research shows that not all extracts/extracted metabolites are cytotoxic to tested BC cell lines (Supplementary Table S1). For example, alliacane sesquiterpenes–purpuracolide B and purpuracolide C extracted from edible fungus *Gomphus purpuraceus* (Iwade) K. Yokoy. 1989 are inactive against MDA-MB-231 cell line (He et al., 2022). This shows how time-consuming and tedious it can be to work on the selection, extraction and testing of a metabolite that may have cytotoxic properties against cancer cells. Another challenge is the selectivity of the natural extracts/extracted metabolites produced in relation to the tested cancer cell lines. For example, Alsaraf et al. (2019) showed that the methanolic extract produced from plantain (*Plantago lanceolata* L., Plantaginaceae) leaves significantly inhibits the proliferation of TNBC CAL51 cells but demonstrate minor effect on the MDA-MB cells (Alsaraf et al., 2019). It also indicates that the heterogenicity of BC or even BC subtypes like TNBC cannot be neglected (Marra et al., 2020).

In the future, it is crucial to conduct comprehensive characterization of the obtained extracts to determine their chemical composition and to identify specific metabolites possessing anticancer properties. Some studies have already conducted fractionation of natural extracts and identified metabolites (e.g., pancratistatin (Youssef et al., 2022), lupeol (Sánchez-Valdeolívar et al., 2020), oleuropein aglycone (Zwartsen et al., 2019; Mazzei et al., 2020;), pyoluteorin (Ding et al., 2020), atranorin (Harikrishnan et al., 2021)) showing potential activity against endocrine-independent HER-2 negative hormone-pretreated BC.

## 4 Pre-clinical data

While clinical guidelines recognize the overlap in features and treatment of TNBC and endocrine-resistant BC (Moy et al., 2021), studies in molecular biology and pharmacology often overlook the issue of endocrine-resistant BC. Consequently, there is a scarcity of pre-clinical models and studies exploring these subtypes, with a predominant focus on TNBC in other available reviews.

In this chapter we provide a perspective on recent advances in the primary literature showing limitations of pre-clinical models. We also stress the significance of investigating endocrine-independent HER-2-negative BC subtypes collectively similarly to the approach in clinical guidelines.

### 4.1 *In vitro* studies

Last years have yielded numerous publications regarding *in vitro* studies on natural metabolite derivatives in TNBC, but also a certain amount of research in HER-2 negative endocrine-pretreated BC. Currently, the majority of promising metabolites are derived from plants (e.g., phytochemicals), but fungi and algae/seaweeds are tempting sources as well (Supplementary Table S1).

#### 4.1.1 Defining cell line models

Defining a proper *in vitro* cell line model for both: TNBC and HER-2 negative endocrine-resistant BC is a challenging and neglected task (Cheng et al., 2022; Soosainathan et al., 2022). Attempts to create hormone-resistant model usually involve growing a cell line (originally from the Luminal BC cell line) that has developed resistance to a particular type of hormone therapy, such as tamoxifen (selective estrogen receptor modulator), fulvestrant (ER antagonist) or aromatase inhibitors. Authors usually focus on inducting hormonal resistance in the commonly used endocrine-sensitive MCF-7 cell line, which only partially mimics real heterogenous clinical scenario (Cardoso et al., 2020; Soosainathan et al., 2022) (Supplementary Table S1). What is more, in case of *in vitro* studies with hormone-pretreated BC cell line models, the efforts are commonly directed at reversing the hormone-resistance using natural metabolites, not at showing potential effects of new chemotherapeutics (Wang et al., 2021). Regarding TNBC cell lines: MDA-MB-231, MDA-MB-468, and CAL51 are most commonly used. Among the cell lines mentioned, MDA-MB-231 are the most frequently applied TNBC model in *in vitro* studies (Supplementary Table S1). Considering high heterogenicity in TNBC (Marra et al., 2020) using a cell line characterized by mutation in V-Raf murine sarcoma viral oncogene homolog B1 (BRAF) and Neurofibromatosis 1 genes does not reflect potential response to the treatment in all TNBC subtypes (Wagner, 2022).

When metabolites are evaluated in conventional cell line models, the scenario parallels the topical application of a ‘candidate medication’, as these compounds do not undergo the full spectrum of Absorption, Distribution, Metabolism, and Excretion (ADME) pharmacokinetic processes.

#### 4.1.2 Improving cell line models

Overall, these *in vitro* models possess numerous limitations. Improved *in vitro* pre-clinical models, such as organoids, organ-on-a-chip, tissue-on-a-chip, bioengineered tissue models and multi-cellular spheroids may hold superiority over simpler patient-derived cell lines for identifying novel classes of drugs. Nevertheless, even with these advancements, these models exhibit significant limitations, including restricted physiological relevance, challenges in standardization and reproducibility and high costs (Xu et al., 2021b; Yadav et al., 2021).

*In vitro* metabolism techniques, which utilize liver fractions or other metabolically capable systems such as primary hepatocytes or recombinant enzymes, represent a significant advancement. These innovative approaches not only facilitate the dissection of biological processes but also accelerate drug development and enhance the efficiency of toxicology studies (Ooka et al., 2020).

What is more, the potential benefit of using novel delivery techniques, e.g., drug nanoform is difficult to be examined in *in vitro* studies as potential benefits of these drugs can result from improved solubility or obtaining more comfortable route of administration.

### 4.2 *In vivo* studies

#### 4.2.1 Currently applied *in vivo* models

*In vivo* studies in mice with anticancer metabolites face numerous limitations in terms of translatability to humans, capturing the complexity of tumor heterogeneity, and accurately reflecting human immune responses. The application of the natural products in mice injected with MDA-MB-231 cells results in decrease in the tumor size/volume and tumor weight (Utage et al., 2018; Collard et al., 2020; Luo et al., 2022). Recently published data from *in vivo* researches suggest that natural products derived from various sources have the potential to inhibit tumor growth, reduce angiogenesis, proliferation, and modulate the tumor microenvironment in TNBC and HER-2 negative hormone-pretreated BC. However, the efficacy and interactions of these natural products may vary. Further research is needed to elucidate the underlying mechanisms and optimize their therapeutic application (Supplementary Table S2).

#### 4.2.2 Improving *in vitro* models

Advanced *in vivo* studies (e.g., patient-derived xenograft models or humanized mouse models) can improve data quality but also come with ethical concerns, limited predictive power and high costs (Xu et al., 2021b; Yadav et al., 2021).

While number of the presented studies can be considered cutting edge research in pre-clinical setting the effectiveness of these chemotherapies as new and highly efficient therapeutic options should be further examined in clinical trials.

## 5 Mechanistic findings for novel naturally-derived drugs

### 5.1 The main mechanisms responsible for the anticancer activity of naturally-derived metabolites

**FIGURE 2:**
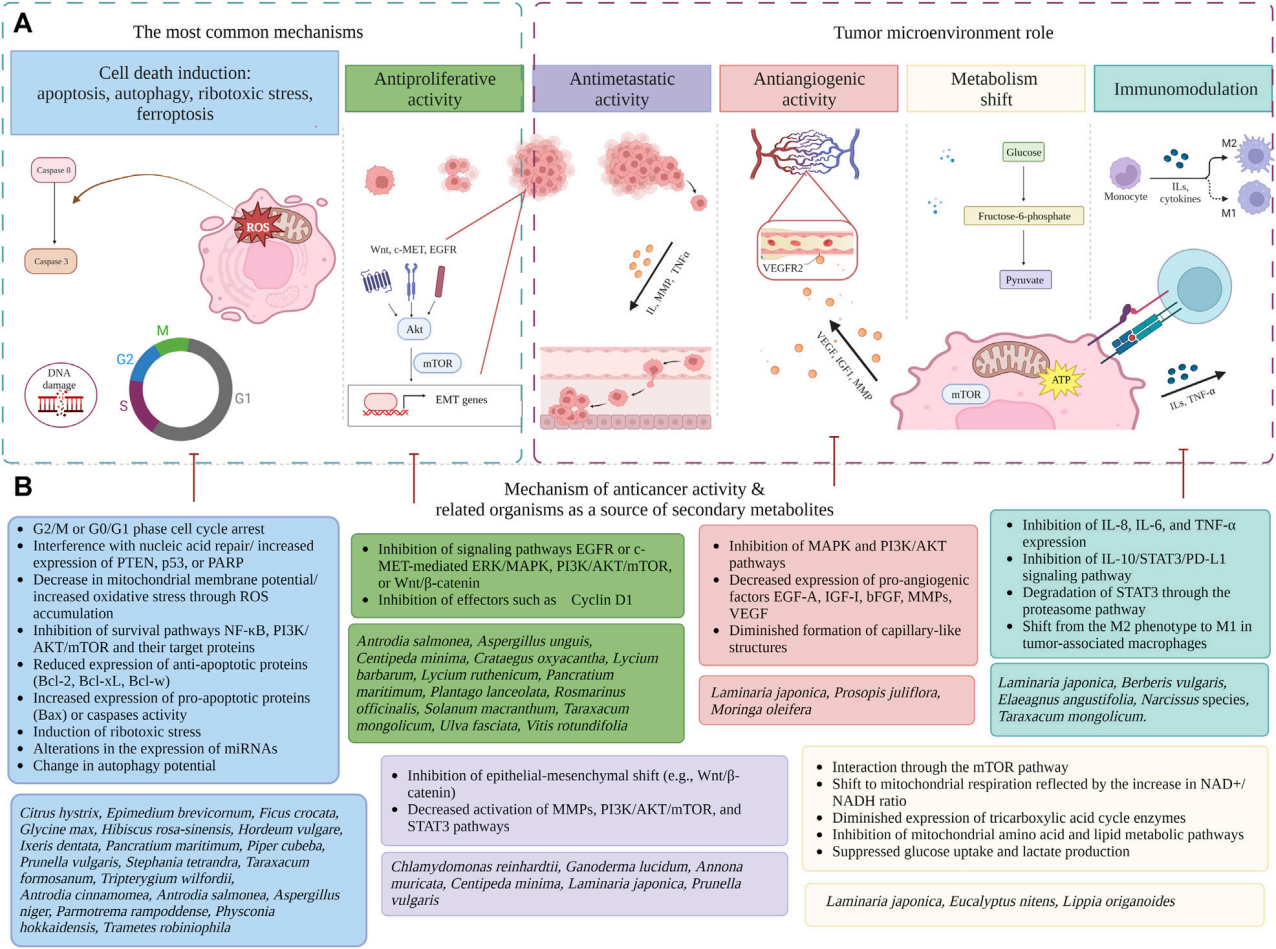
An overview of mechanistic effects of natural metabolites in endocrine-independent HER-2 negative breast cancer (created with BioRender). **(A)** Diagrammatic representation of the mechanistic findings: cell death induction (Cheng et al., 2019; Guo and Pei, 2019; Nguyen et al., 2019; Zhang et al., 2019; Ding et al., 2020; Ho et al., 2020; Liu et al., 2020; Sánchez-Valdeolívar et al., 2020; Harikrishnan et al., 2021; Maungchanburi et al., 2021; Noh et al., 2021; Fouzat et al., 2022; Lin et al., 2022; Luo et al., 2022; Sancha et al., 2022; Sangpairoj et al., 2023), antiproliferative activity (Alsaraf et al., 2019; Zwartsen et al., 2019; Collard et al., 2020; Jaglanian and Tsiani, 2020; Kalebar et al., 2020; Lee et al., 2020; Mazzei et al., 2020; Yang et al., 2021; Lin et al., 2022; Youssef et al., 2022; Kombiyil and Sivasithamparam, 2023) antimetastatic activity (Hsu et al., 2020; Lee et al., 2020; Chen et al., 2022; Luo et al., 2022); antiangiogenic activity (Utage et al., 2018; Hsu et al., 2020; Zunica et al., 2021), disrupted cancer cell metabolism (Raman et al., 2018; Guerra et al., 2021; Yang et al., 2021; Chen et al., 2022; Ke et al., 2022), immunomodulation (Kim et al., 2018; Zhao et al., 2020; 2021; Deng et al., 2021; Chen et al., 2022; Fouzat et al., 2022; Lv et al., 2022). **(B)** Each mechanism of anticancer activity is described in details and recently studied organisms as a source of secondary metabolites are presented (full species names including authorities and family are presented in Table 1; Supplementary Table S1. Abbreviations: AKT, protein kinase B; Bax, Bcl-2-associated X protein; Bcl-2, B-cell lymphoma 2; Bcl-w, B-cell lymphoma 2-like protein; Bcl-xL, B-cell lymphoma-extra-large; BC, Breast cancer; bFGF, basic fibroblast growth factor; CDK, cyclin-dependent kinase; EGFR, epidermal growth factor receptor; EGF-A, epidermal growth factor A; EMT, epithelial mesenchymal transition; ERK, extracellular signal-regulated kinase; HER-2, human epidermal growth factor receptor-2; IGF-I, insulin-like growth factor-I; IL, interleukin; MAPK, mitogen-activated protein kinase; mTOR, mammalian target of rapamycin; miRNA, microRNA; MMP, matrix metalloproteinase; NAD+, nicotinamide adenine dinucleotide (oxidized form); NADH, nicotinamide adenine dinucleotide (reduced form); NF-κB, nuclear factor-kappa B; PARP, poly (ADP-ribose) polymerase; PI3K, phosphatidylinositol-3-kinase; PD-L1, programmed death-ligand 1; p53, tumor protein 53; PTEN, phosphatase and tensin homolog; ROS, reactive oxygen species; STAT3, signal transducer and activator of transcription 3; TNF-α, tumor necrosis factor-alpha; VEGF, vascular endothelial growth factor; Wnt, wingless-related integration site.

An overview of numerous effects of natural metabolites in TNBC and HER-2 negative endocrine-resistant BC indication is presented in Figure 2. Most registered chemotherapeutic agents used in BC treatment act through inhibition of mitosis, lead to cell cycle arrest, influence deoxyribonucleic acid (DNA) replication processes, and thus induce apoptosis through well-known mechanisms (see Table 1).

#### 5.1.1 Cell death induction

Programmed cell death is a broad and intricate concept encompassing a diverse array of mechanisms, including apoptosis, which occurs as a consequence of numerous changes in cancer cells. Some of these mechanisms have been identified for naturally-derived drugs (Alsaraf et al., 2019; Cheng et al., 2019; Guo and Pei, 2019; Mendonca et al., 2019; Nguyen et al., 2019; Zhang et al., 2019; Ding et al., 2020; Ho et al., 2020; Jaglanian and Tsiani, 2020; Lee et al., 2020; Mazzei et al., 2020; Sánchez-Valdeolívar et al., 2020; Harikrishnan et al., 2021; Maungchanburi et al., 2021; Noh et al., 2021; Yang et al., 2021; Fouzat et al., 2022; Ke et al., 2022; Lin et al., 2022; Luo et al., 2022; Sancha et al., 2022; Sangpairoj et al., 2023) (Figure 2). The exact mechanism leading to cell death is essential to be defined as it can predict drugs’ toxicity, efficacy or suggest potential effects of combining with other medications such as synergistic or complementary effect. In a study aimed at assessing the cytotoxic properties of secondary metabolites isolated from *Aspergillus niger* soil fungus (Aspergillaceae), pyoluteorin was observed to arrest the cell cycle of TNBC cells at the G2/M phase. Further experiments suggested that pyoluteorin’s apoptotic effect was linked to a decrease in mitochondrial membrane potential and an accumulation of reactive oxygen species (ROS). (Ding et al., 2020). ROS can react with a variety of biological molecules like DNA, proteins, lipids inducing their damage and initiate apoptosis of cancer cells (Alsaraf et al., 2019; Mendonca et al., 2019). Increased oxidative stress and decreased mitochondrial membrane potential were also observed in *in vitro* and *in vivo* HER-2 negative BC models for the following extracts: the common hibiscus (*Hibiscus rosa-sinensis* L., Malvaceae) (Nguyen et al., 2019), sea daffodil (*Pancratium maritimum* L., Amaryllidaceae) (Sancha et al., 2022), lichen *Parmotrema rampoddense* (Nyl.) Hale 1974, Parmeliaceae (Harikrishnan et al., 2021).

Interfering with the ability to repair nucleic acids is another mechanism leading to the death of cancer cells. Combining cisplatin with triptolide, a natural metabolite derived from a Chinese herb–*Tripterygium wilfordii* Hook. f. (Celastraceae) for treating TNBC caused DNA damage and arrested TNBC cells in the S phase of the cell cycle, making them more sensitive to cisplatin treatment. Triptolide decreased the levels of The Poly (ADP-ribose) polymerase (PARP1) and X-ray repair cross complementing protein 1 (XRCC1), which are involved in repairing single-strand breaks and base excision, on protein level (Zhang et al., 2019).

Inhibition of survival pathways is one of the key mechanisms by which natural metabolites provoke apoptosis. Among these signaling pathways: phosphatidylinositol-4,5-bisphosphate 3-kinase catalytic subunit alpha/protein kinase B (PI3K/AKT) and nuclear factor-kappa B (NF-κB) can be named (Yang et al., 2021; Ke et al., 2022).

The inhibition of anti-apoptotic proteins B-cell lymphoma 2 (Bcl-2) and B-cell lymphoma 2 (Bcl-xL) and activation of pro-apoptotic proteins Bcl-2-associated death promoter (Bad) and Bcl-2-associated X protein (Bax) that regulate mitochondrial pathway and lead to altered caspase pathway expression is the next mechanism. Extract from Kaffir lime (*Citrus hystrix* DC., Rutaceae) leaves contain citronellol and citronellal. These metabolites have been shown to inhibit the expression of Bcl-2 and activate pathway dependent on caspase-3 in TNBC *in vivo* model (Ho et al., 2020). Similar results for caspase-3 activation were observed for *Prunella vulgaris* L. (Lamiaceae) extract (Luo et al., 2022). Another plant extract obtained from red guava (*Psidium guajava* L., Myrtaceae) induced apoptosis through caspase-3, but also through PARP signaling (Liu et al., 2020). Induction of numerous caspases activity was obtained *in vivo* on BC lines with *Piper cubeba* L. f. (Piperaceae) seeds extract (Maungchanburi et al., 2021). The *Elaeagnus angustifolia* L. (Elaeagnaceae) flower extract induced pro-apoptotic proteins such as: Bax and cleaved caspase-8 and reducing the anti-apoptotic Bcl-2 (Fouzat et al., 2022). Alkaloids obtained from sea daffodil (*Pancratium maritimum* L.) also increased Bax expression, and decreased Bcl-xL expression (Sancha et al., 2022). The dichloromethane extract from a tree *Ficus crocata* (Miq.) Mart. ex Miq. (Moraceae) was found to have apoptotic effects on MDA-MB-231 cells, leading to increased expression of tumor suppressor protein p53, as well as procaspase-8, and procaspase-3 (Sánchez-Valdeolívar et al., 2020). It has been found that the treatment with lichen (*Physconia hokkaidensis* Kashiw. 1975; Physciaceae) extract resulted in the downregulation of Bcl-2, p-AKT and adenosine monophosphate-activated protein kinase (AMPK) and while significantly upregulating the levels of cleaved caspase-9, cleaved caspase-3, and cleaved-PARP. *Physconia hokkaidensis* Kashiw. 1975 extract showed selective cytotoxicity toward TNBC lines, but not luminal BC lines (Noh et al., 2021). The extract obtained from common selfheal (*Prunella vulgaris* L., Lamiaceae) triggered cell apoptosis by enhancing nuclear DNA damage and augmenting the expression of cleaved caspase-3 (Luo et al., 2022). The lichen *Parmotrema rampoddense* (Nyl.) Hale 1974 contains a metabolite called atranorin, which has antimicrobial properties, but a study of Harikrishnan et al. (2021) found that atranorin can also have anticancer properties–it significantly decreased the levels of anti-apoptotic proteins such as AKT, Bcl-2, Bcl-xL, and B-cell lymphoma 2-like protein (Bcl-w), while increasing the levels of pro-apoptotic protein Bax and caspases-3 activity in breast cancer cells. This effect was even greater than that of the AKT inhibitor ipatasertib (Harikrishnan et al., 2021). Resveratrol, a polyphenol derived from numerous fruit plants, induced apoptosis in TNBC cells by reducing DNA polymerase delta 1 (POLD1) expression. This mechanism involved decreased levels of anti-apoptotic proteins like Proliferating cell nuclear antigen and BCL-2, increased expression of Cleaved-PARP1 and Cleaved-Caspase3, and potential binding of resveratrol to POLD1 functional domains (Liang et al., 2021).

The metabolite known as oleuropein aglycone was created by using enzymes to break down oleuropein found in olive leaves. This substance was found to have pro-apoptotic effects on two different types of cancer cells: MDA-MB-231 and Tamoxifen-resistant MCF-7 (Mazzei et al., 2020). Icariin, a major metabolite extracted from a Chinese herb *Epimedium brevicornum* Maxim. (Berberidaceae) showed potential to overcome tamoxifen resistance in MCF-7/TAM cells. It induced cell cycle arrest in the G0/G1 phase, apoptosis, and inhibited autophagy. At the molecular level, icariin treatment resulted in the downregulation of CDK2, CDK4, Cyclin D1, Bcl-2, Microtubule-associated protein light chain 3 (LC3-1, LC3-II), Autophagy-related protein 5 (AGT5), and Beclin-1, but upregulated the expression of caspase-3, PARP, and p62 (Cheng et al., 2019). Tetrandrine, an alkaloid extracted from the Chinese herbal medicine–*Stephania tetrandra* S. Moore (Menispermaceae) root targeted autophagy genes LC3-II/LC3-I, p62/SQSTM1, and Beclin-1, inhibited PI3K/AKT/mTOR signaling and increased Phosphatase and tensin homolog (PTEN) expression in TNBC cell lines (Guo and Pei, 2019). The extract obtained from red alga *Halymenia durvillei* Bory de Saint-Vincent (Halymeniaceae) was also found to increase the expression of LC3 (an indicator of autophagic cell death) (Sangpairoj et al., 2023). Extract from dandelion (*Taraxacum formosanum* Kitam., Asteraceae) induced another interesting mechanism that can lead to cell death in BC cell lines: ribotoxic stress. Ribotoxic stress refers to a cellular pathway that reacts to errors in translation, triggering the activation of p38 and c-Jun N-terminal kinases. This response leads to cell cycle arrest, the generation of inflammatory cytokines, and the initiation of apoptotic signaling (Lin et al., 2022). Ferroptosis is an iron-dependent programmed cell death, distinct from apoptosis. It involves the metabolism of unsaturated fatty acids, leading to lipid peroxidation and eventual cell death. This mechanism seems to be caused by both well-known drugs (such as doxorubicin), but also by plant-derived metabolites, e.g., curcumin (Cao et al., 2022; Kciuk et al., 2023).

#### 5.1.2 Antiproliferative and antimetastatic activity

Other important mechanisms include inhibition of cells’ proliferation (Alsaraf et al., 2019; Zwartsen et al., 2019; Collard et al., 2020; Jaglanian and Tsiani, 2020; Kalebar et al., 2020; Lee et al., 2020; Mazzei et al., 2020; Yang et al., 2021; Lin et al., 2022; Youssef et al., 2022; Kombiyil and Sivasithamparam, 2023) and metastatic potential (Avila-Carrasco et al., 2019; Hsu et al., 2020; Lee et al., 2020; Chen et al., 2022; Luo et al., 2022) (Figure 2). Ability to inhibit the growth and division of cancer cells is a hallmark of anti-cancer treatment. By inhibiting cancer cell growth mainly through interfering with signaling pathways, such as the Epidermal growth factor receptor (EGFR)-Mediated Extracellular signal-regulated kinases/Mitogen-activated protein kinases (ERK/MAPK), Wnt/β-catenin or PI3K/AKT/mTOR (partially overlapping with these responsible for proapoptotic, antiangiogenic or antimetastatic potential) natural metaboliteshave the potential to prevent the cancer progression and improve the efficacy of cancer treatments (Cumaoglu et al., 2018; Yang et al., 2021; Kombiyil and Sivasithamparam, 2023). For instance, pre-treatment with extracts derived from shrubs’ *Lycium barbarum* L. and *Lycium ruthenicum* Murray (Solanaceae) fruit has been shown to inhibit the phosphorylation of EGFR and ERK in MDA-MB-231 cells stimulated with epidermal growth factor (Cumaoglu et al., 2018). The methanolic extract of hawthorn (*Crataegus oxyacantha* L., Rosaceae) berry, which is rich in polyphenols, was observed to have toxic effects on TNBC cell lines by exhibiting a significant downregulation of the transcriptional and translational expression of Wnt pathway agonists, as well as an upregulation of Wnt antagonists (Kombiyil and Sivasithamparam, 2023). Seaweed–*Ulva fasciata* Delile (Ulvaceae) has been found to exhibit inhibitory effects on the signaling pathway involving EGFR/PI3K/AKT, resulting in the induction of cytotoxicity in TNBC cells (Pragna Lakshmi et al., 2017).

Muscadine grapes extract has showed its antiproliferative activity *in vitro* and *in vivo* (was given to nude mice with human TNBC tumors for 4 weeks). It decreased TNBC size and reduced the markers Ki67 and cyclin D1 in *in vivo* studies on nude mice and reduced c-Met and inhibited ERK/MAPK and AKT signaling, leading to decreased cyclin D1 and cell cycle arrest in *in vitro* experiments (Collard et al., 2020). Another study has demonstrated that the aqueous extract of *Solanum macranthum* Dunal fruit (the potato tree from Solanaceae) could potentially hinder the growth of MDA-MB-231 cells when it was dissolved in water (Kalebar et al., 2020). The use of *Plantago lanceolata* L. leaf extract on BC cells showed a significant ability to inhibit the growth of CAL51 cells, which are classified as TNBC. However, the extract had only a minor effect on other types of breast cancer cells. Moreover, at higher doses, it caused visible morphological changes to the cells (Alsaraf et al., 2019). Another investigation involved analysis of sea daffodil (*Pancratium maritimum* L.), a member of the Amaryllidaceae, as a potential anticancer agent. Pancratistatin, a bioactive metabolite, was isolated and its growth inhibitory effects were assessed on MDA-MB-231 cells (Youssef et al., 2022). Oleuropein aglycone derived from olive leaves displayed antiproliferative activity against both TNBC MDA-MB-231 and Tamoxifen-resistant MCF-7 call lies (Mazzei et al., 2020). Other natural metabolites that reduced proliferation of TNBC cell lines *in vivo* and *in vitro* were derived from: dandelion (*Taraxacum mongolicum* Hand.-Mazz., Asteraceae) (Lin et al., 2022), fungus *Aspergillus* sp. (Zwartsen et al., 2019), rosemary (*Rosmarinus officinalis* L., Lamiaceae) (Jaglanian and Tsiani, 2020), herb–*Centipeda minima* (L.) A. Braun & Asch., Asteraceae (Lee et al., 2020), or mushroom *Antrodia salmonea* T.T. Chang & W.N. Chou, Polyporaceae (Chang et al., 2017).

The ability to create metastatic disease is one of later steps in cancer development. It requires additional molecular alterations such as epithelial-mesenchymal shift in the metastatic cascade to allow cancer cell to move and develop as metastasis. Among the mechanisms already described in Section 7.4, increased expression of MMPs and disrupted expression of adhesive molecules (e.g., increased N-Catherin, β-Catenin and decreased E-Catherin) are noted (Wilusz and Majka, 2008; Avila-Carrasco et al., 2019). Ebushicao (*Centipeda minima* (L.) A. Braun & Asch.) extract in TNBC cell line led to decreased MMP-9, PI3K/AKT/mTOR and STAT3 pathways activation, resulting in an inhibited metastatic process (Lee et al., 2020). Another extract from medicinal fungus *Ganoderma lucidum* (Curtis) P. Karst (Ganodermataceae) inhibited the release of Interleukins (IL8, IL6), MMP-6 and MMP-9 and reduced TNBC cell migration (Barbieri et al., 2017). Fucoidan from brown seaweed *Laminaria japonica* Areschoug, 1851 (Laminariaceae) can also significantly reduce the ability of TNBC cells to metastasize (Hsu et al., 2020). Antimetastatic activity in this BC setting was also noted for extract derived from: common selfheal (*Prunella vulgaris* L.), green alga *Chlamydomonas reinhardtii* (CC-124) from Chlamydomonadaceae, and soursop (*Annona muricata* Linn) from Annonaceae (Syed Najmuddin et al., 2016; Kamble et al., 2018; Chen et al., 2022; Luo et al., 2022).

### 5.2 Tumor microenvironment role

The tumor microenvironment holds significant importance in pre-clinical research on naturally-derived agents. It consists of a complex interplay of cellular and non-cellular components surrounding the tumor, including immune cells, extracellular matrix, signaling molecules or blood vessels. These factors profoundly influence tumor growth, invasion, and how the tumor responds to various agents.

When exploring mechanisms related to tumor microenvironment role in endocrine-independent HER-2 negative breast cancer antiangiogenic activity (Hsu et al., 2020; Zunica et al., 2021), metabolism shift (Lin et al., 2019; Zhao et al., 2020; 2021; Deng et al., 2021; Chen et al., 2022; Fouzat et al., 2022; Lv et al., 2022) and immunomodulation (Guerra et al., 2021; Yang et al., 2021; Chen et al., 2022; Ke et al., 2022) (Figure 2) are presented.

#### 5.2.1 Antiangiogenic activity

As an example, fucoidan, extracted from brown seaweed *Laminaria japonica* Areschoug, 1851, has been shown to inhibit Mitogen-activated protein kinase and PI3K/AKT pathways, as well as the expression of several pro-angiogenic factors including: Epidermal growth factor A (EGF-A), Insulin-like growth factor I (IGF-I), Basic fibroblast growth factor (bFGF), Metalloproteinases 2 and 9 (MMP-2 and 9) and Vascular endothelial growth factor (VEGF) leading to diminished formation of capillary-like structures (Hsu et al., 2020).

#### 5.2.2 Immunomodulation

Berberine is a plant derivative that influences BC immunity through several mechanisms, including reduced expression of IL-6 and Tumor necrosis factor-α (TNF-α) weakening the inflammatory processes induced by TNBC cell lines (Zhao et al., 2020). Another example is a flowering plant–dandelion (*Taraxacum mongolicum* Hand.-Mazz.) extract that inhibited IL-10/Signal transducer and activator of transcription 3 (STAT3)/PD-L1 signaling pathway as well as shifted from the tumor-associated macrophages phenotype M2 to M1 (Deng et al., 2021). Narciclasine, an antimitotic metabolite derived from daffodil (*Narcissus* L.) bulbs from Amaryllidaceae was able to degrade STAT3 in a specific manner in tamoxifen-resistant MCF-7 cells through the proteasome pathway (Lv et al., 2022).

#### 5.2.3 Metabolism shift

Eucalyptus bark extract used in TNBC cell lines resulted in hydrolysis of cholesterol esters and triglycerides, leading to shift to mitochondrial respiration, which was reflected by increase in Nicotinamide adenine dinucleotide (NAD+, oxidized form) to Nicotinamide adenine dinucleotide (NADH, reduced form) ratio (Guerra et al., 2021). This provides cancer cells with a more efficient energy source and lowers ROS production (Guerra et al., 2021). The extract from the aromatic shrub–*Lippia origanoides* Kunth (Verbenaceae) was found to hinder the metabolic activity of cells by reducing the expression of tricarboxylic acid cycle enzymes and inhibiting mitochondrial amino acid and lipid metabolic pathways. It also disrupted the process of mitochondrial oxidative phosphorylation by inhibiting the expression of multiple subunits of Complex I (Raman et al., 2018).

Further investigation is required to understand the role of the tumor microenvironment and its longitudinal changes during drug treatment, particularly in relation to drug resistance.

### 5.3 Other mechanisms

Natural metabolites effects on cellular or molecular level in BC treatment are also explored in many other aspects than simply using them as potential drugs, e.g., for chemoprotection. One example is protection of hematopoiesis by extract derived from a perennial plant–*Polygonatum sibiricum* Redouté (Liliaceae) (Xie et al., 2021). Polysaccharides contained in this herb are claimed to inhibit activity of myeloid cells in immune tumor infiltrating cells and in spleen (in *in vivo* studies with mice with TNBC) at the same time protecting bone-marrow hematopoiesis (Xie et al., 2021). Other effects include: deactivating the stromal microenvironment by reducing tumor associated fibroblasts (Xu et al., 2020), reversing resistance to chemotherapy (Wang et al., 2020; Yi et al., 2021), and acting on cancer stem cells (Wang et al., 2020; Ke et al., 2022). Some natural metabolites do not act as medications by themselves, but increase sensitivity to currently available treatment options. In the case of hormone-pretreated tumors the efforts are directed at overcoming the endocrine resistance (e.g., Wang et al., 2021). Resveratrol overcomes endocrine resistance in tamoxifen-resistant MCF-7/TR cells by influencing the transforming growth factor-beta (TGF-β)/Smad signaling pathway (Shi et al., 2013). or TNBC tumors studies are aimed at chemotherapy resistance mechanisms (e.g., (Suarez-Arroyo and Martinez-Montemayor, 2018)). Natural raw materials are also known to serve as carriers of many secondary metabolites that have been reported to have anticancer properties in both *in vitro* and *in vivo* experiments, especially metabolites belonging to phenolics, alkaloids, and terpenoids (Püsküllüoğlu and Michalak, 2022).

With all the enthusiasm accompanying these metabolites there is always a risk of increased toxicity or drugs interaction, e.g., in research by Zunica et al. (2021) moringa seed extract (*Moringa oleifera* Lam., Moringaceae) used in *in vivo* experiment on mice with TNBC and obesity has shown a negative interaction when used as a concurrent systemic treatment (Zunica et al., 2021).

## 6 Advancing drug delivery: novel technologies

A rapid advancement within the domain of targeted delivery of cytotoxic agents is observed. Examples of clinically applied strategies in endocrine-independent HER-2-negative breast cancer include nanotechnology and ADCs.

The composition of ADCs presents distinctive hurdles in the characterization of pharmacokinetics and pharmacodynamics. This arises from the necessity for a comprehensive grasp of these processes’ attributes across various molecular entities (such as the conjugate, unbound payload and antibody) within diverse tissues (Li et al., 2020).

### 6.1 Nanotechnology

Nanocarriers offer enhanced drug activity and utility through controlled release, extended circulation, and targeted delivery to cancer cells. Encapsulating drugs in nanoparticles can enable alternative administration routes. These nanocarriers utilize passive and active targeting mechanisms. Among others, nanoforms of taxanes, anthracyclines, camptothecins or eribulin mesylate have recently been tested (Yap et al., 2021; Miguel et al., 2022; Püsküllüoğlu and Michalak, 2022). Nanoparticles can be obtained from natural sources by themselves acting as immunomodulators, drugs, or in cancer prevention or cytoprotection (Püsküllüoğlu and Michalak, 2022). Next to clinically applied nanoforms of drugs new options are tested in pre-clinical and clinical setting (see below). Example of recently published pre-clinical study involves doxorubicin-loaded magnetite nanoparticles in TNBC *in vitro* cell line model (Markhulia et al., 2023).

### 6.2 Antibody-drug conjugates

Currently, the greatest group of targeting agents seems to be ADCs with SG and T-DXd being already approved in this indication (Cardoso et al., 2020; Püsküllüoğlu et al., 2023). ADC selectively recognizes and binds to specific cancer cells. The linker acts as a bridge, connecting the antibody to the cytotoxic payload, which exerts its effect by inducing cell death in the targeted cancer cells. The phenomenon where neighbouring cancer cells, not directly targeted by the drug, are still affected by the cytotoxic payload is called bystander effect. Ideal ADC payloads should have high cytotoxicity, low immunogenicity, and high stability. They should also possess modifiable functional groups for conjugation, promote bystander killing effects, have proper water solubility, and target intracellular sites for effective tumor cell penetration (Püsküllüoğlu et al., 2023; Wang et al., 2023). ADCs including naturally-derived agents that are tested in clinical trials in endocrine-independent HER-2-negative BC are presented in the next chapter. For pre-clinical setting a good example is ADC consisting of humanized MUC1 antibody linked to monomethyl auristatin (MMAE) and showing activity in cell line model (Li et al., 2023). MMAE is an antimitotic agent derived from sea hare (*Dolabella auricularia* [Lightfoot], 1786) from Aplysiidae that is too toxic to be used on its own, but linking to mAb allows toxicity to be limited (Püsküllüoğlu and Michalak, 2022).

It is expected that both: new receptors and new natural metabolites linked to targeting part will be further explored in the nearest future. The emphasis will be placed on the exploration of more specific targets for these BC populations. Exosome-based carriers are another alternative for modern drug delivery system (Song et al., 2021). However, the implementation of exosomes in BC clinical settings has not yet been realized, resulting in an uncertain assessment of their usefulness. Examples of other options used to target cytotoxic agents include targeted small molecules (Sun et al., 2021).

**FIGURE 3:**
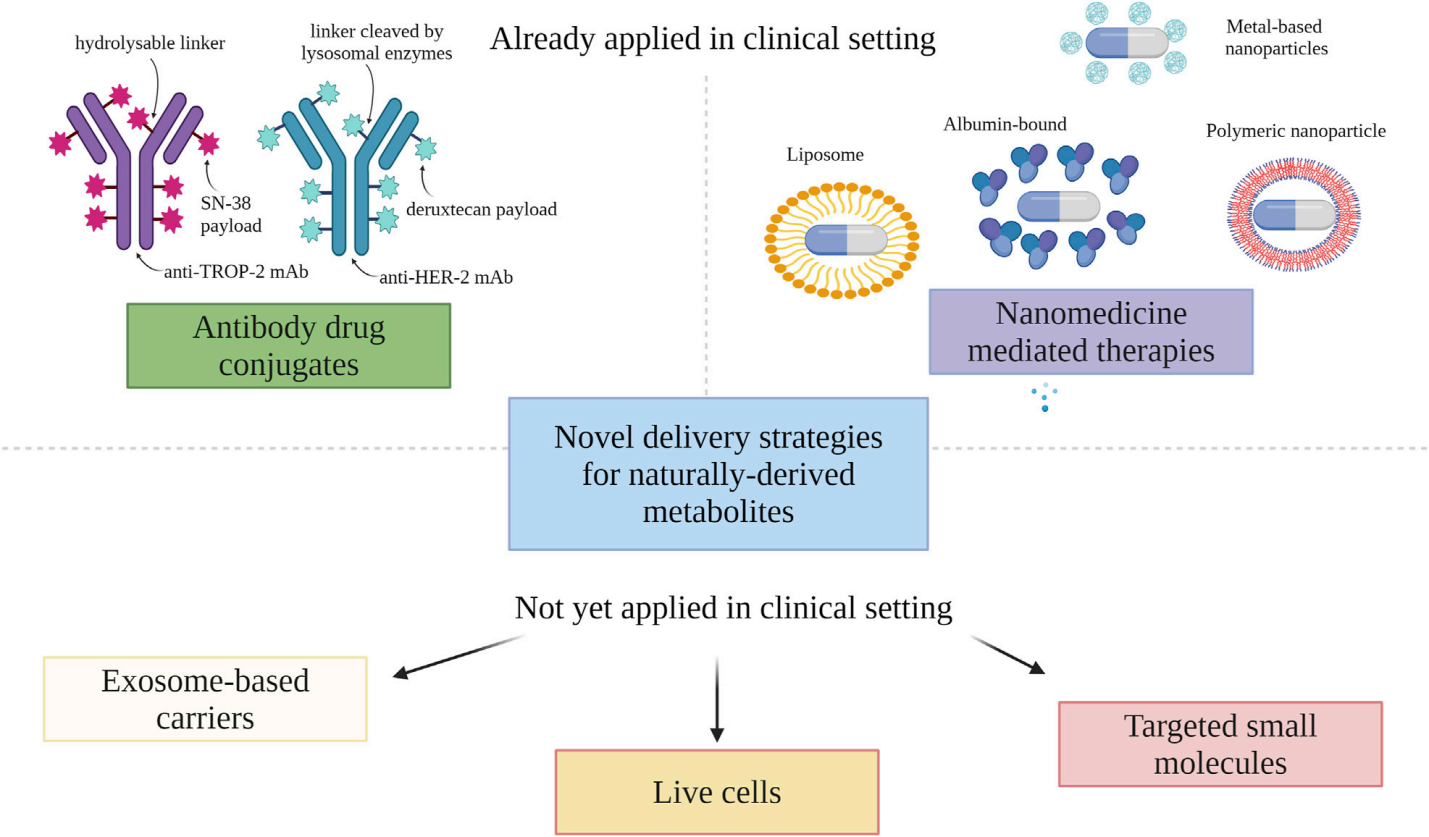
Novel mechanisms for drug delivery (created with BioRender). Abbreviations: HER-2, human epidermal growth factor receptor 2; mAb, monoclonal antibody; TROP2, trophoblast cell surface antigen 2.

Figure 3 presents an overview of already applied and potential delivery strategies for natural metabolites in endocrine-independent HER-2 negative BC.

## 7 Trends in clinical trials

Clinical trials are crucial in drug development, even for effective drugs in pre-clinical studies. They verify safety, efficacy, optimal dosage, and long-term effects in diverse human populations. Due to the pharmacokinetic and pharmacodynamic processes of naturally-derived compounds administered orally or parenterally, their biological effects on breast cancer cells and observed toxicity in the human body can be associated with the activity of their metabolites. Predicting such outcomes in pre-clinical trials is sometimes impracticable. Ethical and regulatory compliance mandates their use before widespread adoption. This section gives authors perspective on trends in ongoing clinical trials in endocrine-independent HER-2 negative breast cancer together with comments regarding trials’ limitations and challenges. Currently, studies are organized around four aspects: checking the safety and efficacy of new naturally-derived metabolites; exploring the potential of drugs of natural origin nanoforms or targeting them through systems such as ADC; investigating new schemes, routes of administration or treatment sequences of already approved drugs with natural origin. In contrast to the pre-clinical scenery, it is easier to select and define hormone-resistant setting in clinical trials as patients’ populations are clearly defined in inclusion and exclusion criteria within the study protocols. Clinical trials, viewed as experiments involving human subjects, are subject to a significantly higher degree of regulations and legal safeguards compared to pre-clinical studies. These include obligations related to their registration (all trials discussed in this section were registered in ClinicalTrials.gov and accessed on 4th November 2023). Clinical trials are conducted in pre-defined phases, during which safety, efficacy, and efficacy in comparison to the existing standard of care are evaluated in a given indication. Despite these regulations ensuring high standards and confidence in the quality of results, the interpretation of clinical trial outcomes can be challenging. For example, unplanned subgroup analyses may provide a misleading sense of excessive certainty regarding the quality of the obtained data. Furthermore, each type of study poses specific challenges, as discussed in each section below.

### 7.1 Trials regarding novel metabolites

Undoubtedly, the highest expectations are placed upon this particular group of studies. The pursuit of novel pharmaceutical agents exhibiting no cross-resistance with previously employed treatments and enhancing overall survival stands as the paramount hope in the realm of clinical research. Challenges may include lack of production standardization, limited access to natural sources, and thus, cost of the treatment. Some naturally-derived medications even when tested in clinical trials have no clear definition of their components or the solvent. Efforts to better control this issue are imperative, as it can lead to challenges in reproducing clinical data, as well as pose a significant risk of drug interactions. NCT04403529is the phase 3 randomized, double-blind trial checking the efficacy of Traditional Chinese Medicine in TNBC adjuvant setting. Primary endpoints are disease free survival (DFS) and quality of life. Unfortunately, clinicaltrial.gov web page devoted to that study does not explain which twelve herbal metabolites are incorporated into that medicine. That issue is a certain limitation in few ongoing clinical trials. In the phase 4 NCT02615457 trial, Huaier granules are being evaluated for their efficacy in radically treated TNBC. Huaier granules are a traditional Chinese medication extracted from a mushroom *Trametes robiniophila* Murrill 1907 (Polyporaceae) applied in colitis, nephrosis, tuberous sclerosis, and various cancers (Narayanan et al., 2023). Following international guidelines all tumors greater than 5 mm should receive preoperative systemic treatment (NCCN Clinical Practice Guidelines in Oncology, 2023). As per protocol patients who completed neoadjuvant treatment are excluded. As a result, a very low recruitment rate or comparison to control arm receiving the treatment inferior to current standard of care can be expected. This is another limitation of these clinical trials. The phase 1/2 NCT03387085 trial in metastatic TNBC setting tests metronomic chemotherapy of numerous naturally derived agents in terms of safety and ORR. Drugs such as: albumin-binding prodrug of doxorubicin; a set of vaccines derived from recombinant *Saccharomyces cerevisiae* yeast; nab-paclitaxel and other chemotherapeutics and biological agents are included in that trial. The phase 2 NCT05007444 study with P2Et (a standardized extract of tara (*Caesalpinia spinosa* (Molina) Kuntze, Fabaceae) in addition to standard neoadjuvant therapy is being performed on BC patients (not eligible for anti-HER-2 treatment) in order to assess optimal biological dose of P2Et based on its toxicological profile and other parameters as secondary endpoints. The phase 2 NCT05403333 trial is examining the efficacy (PFS) of weekly utidelone in HER-2 negative inoperable or metastatic BC (both TNBC and hormone-pretreated). This metabolite, a modified epothilone (see Table 1 for ixabepilone source) analog has already been tested in BC setting and although positive, the previous study did not result in drug approval (Xu et al., 2021a). An intriguing study, yet one that raises many unanswered questions and concerns (e.g., about the broad study population, which includes DCIS, and dosage, staging of the disease), is the phase 1 trial (NCT05680662) examining herbal titled “The Study of Quadruple Therapy Quercetin, Zinc, Metformin, and EGCG as Adjuvant Therapy for Early, Metastatic Breast Cancer, and Triple-negative Breast Cancer”. Quercetin, present in foods like kale, berries, onions, cherries, red grapes, broccoli, tea, and red wine, is traditionally used to prevent diseases such as osteoporosis, lung and cardiovascular diseases, and cancer. Epigallocatechin gallate (EGCG) is a polyphenol present in green tea has been tested for its chemopreventive activity (Du et al., 2012; Xu et al., 2019). Natural metabolites have been also tested in other contexts such as primary or secondary BC prevention and management of side effects. Epidiferphane, a claimed neuroregenerative dietary supplement (Steindler and Reynolds, 2017), is being evaluated alongside taxanes in BC to prevent peripheral neuropathy in the phase 1/2 NCT05074290 trial. Pentoxifylline, a synthetic xanthine derivative originally derived from coffee beans and tea leaves (Dastgheib et al., 2022). Unfortunately, most studies in this context are expected to yield negative results, as seen with soy protein for BC prevention in the NCT00204477 trial or PSC 833 (a cyclosporine derivative from the fungus *Tolypocladium inflatum* W. Gams 1971, Ophiocordycipitaceae) for multidrug-resistance inhibition in BC patients receiving taxanes (NCT00002826).

### 7.2 Trials regarding drugs’ nanoforms

Studies regarding nanoforms of existing medications and drugs that are active, but too toxic to be used on their own are costly and more likely to fail than traditional ones. What is more, the benefits of introducing drug nanoform are difficult to prove in pre-clinical studies. Nonetheless, that direction is constantly proving to be effective in terms of obtaining better treatment outcomes, diminishing toxicity, offers better solubility or a new route for administration (Püsküllüoğlu and Michalak, 2022). In a single arm phase 2 the PHENOMENAL study (NCT03328884) nanoliposomal irinotecan is being tested for central nervous system (CNS) ORR in HER-2 negative BC patients with brain metastases. This drug is also being evaluated in combination with pembrolizumab in TNBC brain metastatic population in phase 2 NCT05255666 trial with CNS Disease Control Rate (DCR) as the primary endpoint. While nanoforms of irinotecan are already approved for use in other indications such as pancreatic cancer (Wang-Gillam et al., 2019) docetaxel’s nanoforms have no registration as of now. In phase 3 NCT03671044 study, nanosomal docetaxel lipid suspension and a standard docetaxel are compared for ORRs in later lines of palliative TNBC treatment. Paclitaxel’s nanoforms have been approved for use in few indications including metastatic BC or pancreatic cancer (Samaan et al., 2019). In phase 3 NCT04137653 study, nab-paclitaxel is being tested in radical treatment of TNBC.

### 7.3 Trials regarding drugs linked to targeting part

Among potential benefits of ADC: selective toxicity, increased potency, reduced drug resistance can be named. The limitations in these trials are an industry-sponsored bias and a risk of setting endpoints that is not optimal, but easier to achieve. Additionally, endocrine-independent HER-2-negative breast cancer lacks specific therapeutic targets tailored to this subtype, potentially limiting the efficacy of tested ADCs. Patritumab Deruxtecan is a camptothecin derivative linked to anti-HER-3 mAb. In the phase 2 NCT04699630 trial, ORR and 6-month PFS are being assessed in metastatic BC including two arms for HER-2 negative endocrine-resistant patients. In another phase 2 NCT04965766 trial, the same drug is being tested for ORR in the metastatic HER-2 negative endocrine-resistant BC population. The phase 3 ANGLeD trial (NCT03613181) covers HER-2 negative metastatic BC patients with leptomeningeal disease and extremely unfavorable prognosis. Paclitaxel trevatide (paclitaxel-Angiopep-2 conjugate) is compared to other agents (including eribulin mesylate) to check for OS. In the phase 2 NCT04742153 trial MR002, an anti-HER2 mAb conjugated MMAE is being assessed for ORR in HER-2 low metastatic BC. The phase 1/2 NCT04441099 trial assesses the dose and anti-tumor activity of anthracycline-based ADC targeting Receptor tyrosine kinase-like orphan receptor 1 in few indications including metastatic TNBC (for the phase 2 part). The TROPION-PanTumor02 phase 1/2 (NCT05460273) trial explores the anti-TROP2 mAb linked to deruxtecan ADC (Datopotamab- DXd) in terms of ORR in different BC cohorts.

### 7.4 Trials with currently approved agents

In number of trials, currently approved drugs are being tested to search for better administration scheme or set standards in their combination with other medicines or determine optimal therapy sequences. The main limitation is the retrospective observational design of these studies, driven by challenges in organizing randomized controlled trials (RCTs) for non-patented drugs, primarily due to financial constraints. Metronomic chemotherapy uses low doses of drugs at regular short intervals, with antiangiogenic and antineoplastic effects (Cazzaniga et al., 2021). For example, the phase 2 NCT03071926 trial explores the outcomes of metronomic pegylated doxorubicin in endocrine-resistant advanced BC, while phase 2 NCT05747326 study checks 1-year performance free survival (PFS) of capecitabine and vinorelbine in HER-2 negative BC. An example of study that tries to set the place for commonly used drugs in TNBC radical treatment setting is phase 4 NCT04136782 trial with carboplatin, nab-paclitaxel, epirubicin and docetaxel.

Among the studies exploring new combinations of known drugs, there are numerous examples: the KEYDOX phase 1/2 trial (NCT03591276) studies the efficacy–objective response rate (ORR) and safety of pembrolizumab (an anti-PD-1 mAb) with pegylated liposomal doxorubicin in endocrine-resistant BC. In NCT04039230 phase 1/2 study SG is tested in combination with talazoparib (a PARP inhibitor) in metastatic TNBC. The same ADC is tested together with PI3K inhibitor alpelisib in metastatic, pretreated HER-2 negative BC in the phase 1 ASSET trial (NCT05143229), and other ADC T-DXd is checked for its safety when administered with nivolumab (an anti-PD1 mAb) in phase 1 NCT03523572 study including patients with BC.

Improving clinical trials involving natural-source medications is crucial due to challenges like lack of standardization and limited access to natural sources. Ambiguity in drug components and solvents can hinder data reproducibility and pose drug interaction risks. Addressing these issues through rigorous quality control, standardized protocols, and drug characterization is imperative for more reliable and effective clinical trial outcomes. Improving clinical trials for nanoforms of existing medications and toxic drugs requires addressing cost challenges and optimizing pre-clinical data to enhance the chances of developing safe and effective nanoform treatments. Enhancing clinical studies for drugs composed of natural-source-derived metabolites attached to targeting part requires addressing industry-sponsored bias and carefully selecting clinically relevant endpoints. Transparent trial design, independent oversight, and adaptive approaches can enhance scientific validity and yield more reliable results. Collaborative funding, innovative trial designs, and real-world data utilization are potential approaches to address limitations in clinical trials for currently approved drugs when searching for better therapy sequence, administration scheme or drugs’ combinations.

## 8 Future directions

Endocrine-independent HER-2-negative breast cancer is a condition known for its unfavorable treatment outcomes. Despite the introduction of innovative therapies like immune checkpoint inhibitors, the improvement in patients’ survival remains unsatisfactory. As a result, chemotherapy, primarily based on naturally-derived metabolites, remains the cornerstone of treatment. There are numerous novel natural agents with suggested pre-clinical and currently tested clinical activity in this BC subtype.

• Pre-clinical *in vitro* and *in vivo* models used to assess efficacy of the tested metabolites are far from perfection. The issue lies in the usage of archaic models, such as patient-derived cell lines. Additionally, it would be an overstatement to consider currently available cell lines as good representatives for all TNBCs or for endocrine-resistant HER-2-negative BCs. Regarding TNBC, data are primarily based on 1 cell line, MDA-MB-231. Cell lines obtained from endocrine-pretreated samples also do not adequately reflect numerous possible clinical scenarios. It is essential to employ contemporary pre-clinical models to accurately study the mechanisms, especially in the context of tumor microenvironment and therapeutic effects of naturally-derived agents. Advanced three-dimensional cell culture models or organoid cultures offer relevant platforms for examining how these agents interact with the organism and how they affect disease progression. These advanced models closely mimic the tumor microenvironment and provide a more precise reflection of how individual patients may respond to these treatments.
• Defining the composition of medications, especially if they are mixtures of different ingredients, including the material in which they are dissolved. Some traditional medications even when tested in clinical trials have no clear definition of their components. this can result not only in difficult pre-clinical or clinical data reproducibility, but also generates a risk of drug interactions. Such risk is very high in cancer patients due to the numerous other agents they take, including other anticancer treatment, side effects prevention and treatment, comorbidities treatment or supplements intake (van Leeuwen et al., 2011).
• Biomarkers are quantifiable indicators, including genetic mutations or other molecular features, which furnish valuable insights into the treatment response of a disease. Biomarkers aid in identifying disease subtypes and forecasting individual patient responses to particular naturally-derived therapies. Their pivotal role lies in customizing personalized treatment approaches, empowering healthcare providers to make well-informed decisions and enhance therapeutic outcomes. Investigating predictive factors for new anticancer drugs enhances treatment effectiveness. In pre-clinical research, biomarkers help assess treatment effectiveness and safety in cell and animal studies. For instance, monitoring changes in tumor growth-related biomarkers provides insights into the agents’ mechanisms of action. Each successful pre-clinical concept should be followed by clinical trials, preferably incorporating biomarker analysis in the trial design or planning additional trials to explore specific patients’ groups, rather than conducting post-hoc analyses. The final stage should involve validating biomarkers/predictive factors through appropriate tests applicable in clinical practice (Hughes et al., 2011; Califf, 2018; Berdigaliyev and Aljofan, 2020; Choudhari et al., 2020; Kaushik et al., 2021). Currently, the available treatment options for endocrine-independent, HER-2-negative breast cancer, except for immune checkpoint inhibitors in metastatic TNBC and HER-2 low status for T-DXd lack validated predictive factors similar to those for anti-HER2 treatment. The absence of specific therapeutic targets for this breast cancer subtype may limit the efficacy, even if an agent is associated with targeting mechanism.
• Performing clinical trials and patenting natural metabolites with anticancer activity requires extensive and time-consuming research. Numerous trials are ongoing, but successful studies in this area are relatively few, and patenting issues can further complicate financing for costly clinical trials (Sahoo et al., 2022). Therefore, we advocate for the promotion of increased support for non-sponsored trials.

In summary, current treatment options offer insufficient outcomes for patients with endocrine-independent HER-2-negative breast cancer. There are favorable prospects for the advancement of new naturally-derived drugs in this setting. Nature is still an inexhaustible source of medications, their prototypes, as well as an inspiration for medicines created in the laboratory. Naturally-derived metabolites that have already been rejected due to solubility issues or toxicity profile can come back into play by using new methods of their targeting and delivery such as ADCs or creating their nanoforms. Furthermore, certain studies have shown the synergistic impacts of combining naturally-derived agents with standard therapies, suggesting new approaches to overcome drug resistance and improve treatment outcomes in this complex subgroup of breast cancer. Considerable efforts are yet to be undertaken in unraveling the intricacies of how effectively bring naturally-derived metabolites from bench to bedside.
