# Supplementary material for: The therapeutic potential of natural metabolites in targeting endocrine-independent HER-2-negative breast cancer
Source: Front Pharmacol. 2024 Mar 4;15:1349242. doi: 10.3389/fphar.2024.1349242 (PMC10944949; doi:10.3389/fphar.2024.1349242)
Supplement: Supplementary file 1 [file Table1.docx]

Supplementary Materials

# Supplementary Tables

**Table S1.** The recent examples of *in vitro* investigations with natural products in the triple negative breast cancer and HER-2 negative hormone-pretreated breast cancer models:

| **Type of natural product** | **Production method** | **Potential active metabolites** | ***In vitro* effect of extract on breast cancer cell lines** | **Ref.** |
| --- | --- | --- | --- | --- |
| **Fruit plants** | | | | |
| Muscadine grape berry  (*Muscadinia*  *rotundifolia* Michx, Vitaceae) | Extraction of frozen in liquid nitrogen and powdered whole berries (seeds, skin, pulp) with methanol, shaking, 24 h, room temp., solvent removal – rotary evaporation | Phenolics – flavonoids, antioxidant activity – DPPH^1^ assay | - A dose-dependent (1, 2, 3, 4 µg/µL) cytotoxic effect of extract on MDA-MB-231 with the IC_50_^2^ ranging from 2.7 to 3.7 µg/µL; on MDA-MB-468 from 2.8 to 4.7 µg/µL; - Cytotoxicity of extracts up to 78.6% in MDA-MB-231 and 90.7% in MDA-MB-468 cells; - Strong correlation between total phenolic content and anticancer/antioxidant activity | (Mendonca et al., 2019) |
| Kaffir lime (*Citrus hystrix* DC., Rutaceae) | Extraction (3 ×) of leaf fine powder with hexane or ethyl acetate or ethanol, continuous stirring for 3 days, room temp., filtration, solvent rotary evaporation | Acyclic monoterpenoids: citronellol, citronellal | - Hexane extract reduced cell viability of MDA-MB-231 cells with the IC_50_ of 318±2 µg/mL, IC_50_ of 547±1 µg/mL for ethyl acetate extract and IC_50_>1000 µg/mL for ethanolic extract; - Significant reduction in MDA-MB-231 cells proliferation in a dose (50, 100, 150 µg/mL)- and time (24 h and 48 h)-dependent manner for hexane extract – decrease in % cell viability with increase in extract concentration; - Significant reduction in colony formation of MDA-MB-231 cells in a dose-dependent manner for hexane extract (especially for 100, 150 µg/mL) | (Ho et al., 2020) |
| Red guava  (*Psidium guajava* L.,  Myrtaceae) | Production of a crude juice from the red guava fruit in the mature stage,  grounding of flesh with a juice machine, centrifugation, filtration | Essential oil –  sesquiterpene (β-caryophyllene oxide), triterpenoid acids; phenolics: tannins, flavonoids; vitamins | - Both extracts – total extract and lower molecular weight (<30 kDa^3^) extract (LMW extract) – decreased significantly the viability of the MDA-MB-231 and MDA-MB-468 cells; both extracts induced cytotoxicity in TNBC^4^ cells; - The percentages of necrosis and apoptosis in MDA-MB-231 cells treated with total extract (31.4% necrosis and 9.4% apoptosis) or LMW extract (24.4% necrosis and 7.6% apoptosis) were higher than those in control cells (1.7% necrosis and 0.3% apoptosis) | (Liu et al., 2020) |
| Goji berry red (*Lycium barbarum* L.), and black fruit (*Lycium ruthenicum* Murray),  Solanaceae | Extraction of frozen fruits of red (GBRFE) and black goji berries (GBBFE) with methanol, room temp., three days, filtration | Polysaccharides; tetraterpenoids –  carotenoids; phenolics: gallic acid, *p*-coumaric acid, chlorogenic acid, flavonoid (kaempferol) | - Significant dose-dependent inhibitory effect of GBBFE and GBRFE at 25 to 150 μg/mL on the proliferation of MDA-MB-231 cells; - IC_50_ of 87.0 μg/mL for the GBBFE, IC_50_ of 79.4 μg/mL for GBRFE, IC_50_ of 33.2 μM for cis-platin (positive control) | (Cumaoglu et al., 2018) |
| Hawthorn (*Crataegus oxyacantha* L.,  Rosaceae) | Extraction of powdered berries in 70% methanol by soxhlation, 65 °C 6 h, solvent rotary evaporation, lyophilization of remaining portion | Phenolics: catechin, epicatechin, catechin-o-gallate, epicatechin-o-gallate, quercetin, ellagic acid, gallic acid | - Cytotoxicity of extract to MDA-MB-231 cells with the IC_50_ of 75 μg/mL (tested concentration range from 10 to 1000 μg/mL); - Inhibition of cells proliferation – a significant decrease in the number and size of colonies after 48 h; - Morphological changes caused by extract in MDA-MB-231 cells (cell shrinkage, membrane blebbing, rounding up, anoikis); - Inhibition of cell cycle by extract phytochemicals (with G1/S arrest) in MDA-MB-231 cells | (Kombiyil and Sivasithamparam, 2023) |
| The potato tree (*Solanum macranthum* Dunal,  Solanaceae) | Sequential extraction of dried fruit and fruit calyx separately using four different solvents: petroleum ether, chloroform, methanol, and water, 144 h, filtration, drying | Phenols, alkaloids, saponins | - Cytotoxic effect of aqueous extract (selected from the obtained extracts) on MDA-MB-231 cell line with inhibitory activity of 99.1, 94.5, 81.6, 67.0, 54.1% for extract at 100, 200, 300, 400 and 500 μg/mL, respectively; - At low concentrations (50 μg/mL), the aqueous extract was found to be highly effective at reducing the growth of the MDA-MB-231 cell line | (Kalebar et al., 2020) |
| Resveratrol  obtained from numerous species | Resveratrol – commercially available, production method not stated | A non-flavonoid polyphenol | - Resveratrol reduced the survival of MDA-MB-231 and MCF-10A cells; - The optimal concentration of 50 µM demonstrated a concentration-dependent effect; - RNA sequencing analysis revealed 8,527 differentially expressed genes in MDA-MB-231 cells treated with resveratrol; - Apoptosis-related genes were upregulated, confirmed by Western Blot; - POLD1^5^ expression decreased in cells treated with resveratrol;   Overexpression of POLD1 mitigated the resveratrol-induced apoptosis | (Liang et al., 2021) |

| **Type of natural product** | **Production method** | **Potential active metabolites** | ***In vitro* effect of extract on breast cancer cell lines** | **Ref.** |
| --- | --- | --- | --- | --- |
| **Herbs** | | | | |
| Rosemary (*Rosmarinus officinalis* L.,  Lamiaceae) | Maceration of dried and ground leaves overnight (16 h) in dichloromethane:methanol (1:1), filtration, boiling of leaves in methanol for 30 min, mixing of the solvent after boiling with the filtered solvent, solvent rotary evaporation | Polyphenol: rosmarinic acid; diterpenes: carnosic acid, carnosol | - A dose-dependent inhibition of MDA-MB-231 cells proliferation by rosemary extract; - For 50 µg/mL of extract, max. inhibition of cell proliferation (34.8% of control) – greater than for 10 nM of paclitaxel (72.3% of control); IC_50_ of cell proliferation was 28.9 µg/mL; - For 25 µg/mL of extract, the greatest inhibition (9.33% of control) of cell survival; IC_50_ of cell survival was 4.82 µg/mL – for 2 nM of paclitaxel, inhibition of cell survival was 47.3% of control, for 5 mM of metformin, 63.4% of control | (Jaglanian and Tsiani, 2020) |
| Ebushicao (*Centipeda minima* L. A.Braun & Asch.,  Asteraceae) | Extraction (twice) of powdered sample with 50% ethanol, sonication, room temp., 30 min, centrifugation | Phenolic acids (e.g., isochlorogenic acid A, isochlorogenic acid C); flavonoids; sesquiterpene lactones (brevilin A, arnicolide D) | - A dose (0.25, 0.5, 1, 2.5, 5, 10, 25, 50, 100 μg/mL)- and time-dependent cytotoxic effect of extract on MDA-MB-231 cells; the IC_50_ of extract in MDA-MB-231 cells of 20.0 μg/mL, 12.0 μg/mL, and 5.55 μg/mL, respectively after 24, 48, and 72 h of treatment; - Inhibition of cell proliferation and colony formation after 14 days at doses higher than 1.25 μg/mL (2.5 and 5 μg/mL); - Induction of MDA-MB-231 cells apoptosis (starting from a dose of 10 μg/mL) | (Lee et al., 2020) |
| Tailed paper (*Piper cubeba* L., Piperaceae) – aromatic spice | Extraction of powdered seeds by soaking in dichloromethane (fraction D), and then the solid residue in methanol – M (fraction RD), filtration, solvent rotary evaporation | Total phenolics (Folin-Ciocalteu assay); condensed tannins (proanthocyanidins), flavonoids;  antioxidant activity – DPPH assay | - The strongest cytotoxic effect of extract fraction DE15 (eluted with dichloromethane:methanol, 20:80) against MDA-MB-231 cells with the IC_50_ of 4.43±0.16 μg/mL; for doxorubicin (positive control) the IC_50_ of 2.71±0.13 μM; - Cytotoxic activity of fraction D against MDA-MB-468 cells with the IC_50_ of 23.8±4.1 μg/mL, for fraction RD-M, the IC_50_ of 32.0±0.2 μg/mL; for fraction DE15, the IC_50_ of 6.59±1.08 μg/mL; - Induction of MDA-MB-231 cells apoptosis – for the treatment of cells with the fraction DE15 for 48 and 72 h, the percentage of late apoptosis/dead significantly increased to 37.8% and 37.0% – by the stimulation of multi-caspases activity | (Maungchanburi et al., 2021) |
| A perennial herb – *Prunella vulgaris* L. (PV),  Lamiaceae | Extraction (3 ×) of the powdered material with boiling water, 1 h each (m/v, 1/10), filtration, solvent rotary evaporation  PV20, PV50 and PV95 – 20%, 50% and 95% ethanol eluted fraction | PV20: salviaflaside, rosmarinic acid isomers, phenolic acid; PV50:  rosmarinic acid; PV95: polar triterpenoid components (ursolic acid, oleanolic acid) | - A small proliferation inhibitory effect of PV20 on MDA-MB-231 cells; a strong inhibitory effect of PV50 on cells proliferation with the IC_50_ of 331±7 μg/mL; the strongest inhibitory effect of crude extract and PV95 fraction with the IC_50_ of 73.3±7.5 μg/mL | (Luo et al., 2022) |
| Chinese herb –  *Epimedium brevicornum* Maxim.,  Berberidaceae | Production method not indicated | Icariin – a natural flavonoid glycoside | - Decrease in viability of tamoxifen-resistant breast cancer cell line (MCF-7/TAM) in a dose-(10, 25, 50, 75 μM) dependent manner for 24 h; IC_50_ of about 50 μM against this cell line; - Increase in LDH^6^ activity in MCF-7/TAM cells in a dose-dependent manner; - Significant induction of cell cycle G0/G1 phase arrest, apoptosis, autophagy suppression; - Reduction of autophagic vacuoles in icariin-treated cells compared to control cells | (Cheng et al., 2019) |
| Curcumin derived from *Curcuma longa* L., Zingiberaceae | Curcumin – commercially available | Curcumin – a natural polyphenol | - Suppression in the viability of MDA-MB-453 cell in a dose-dependent manner; - Induction of ferroptosis in MDA-MB-453 cells through the facilitation of solute carrier family 1 member 5; - An increase in lipid ROS^7^ levels, accumulation of the lipid peroxidation end-product malondialdehyde, and elevated intracellular Fe^2+^ levels. | (Cao et al., 2022) |

| **Type of natural product** | **Production method** | **Potential active metabolites** | ***In vitro* effect of extract on breast cancer cell lines** | **Ref.** |
| --- | --- | --- | --- | --- |
| **Flowering plants** | | | | |
| Hibiscus flower (*Hibiscus rosa-sinensis* L.,  Malvaceae) | Extraction of a fine powder in boiled double distilled water, 60 °C, 3 h, isolation through a cheese cloth | Phytochemicals | - The combination of hibiscus extract at 1 mg/mL with chemotherapeutics (taxol and cisplatin) significantly increased the induction of apoptosis when compared to chemotherapeutic treatments alone (by an increase in ROS production and a breakdown of the mitochondrial membrane) | (Nguyen et al., 2019) |
| Ribwort/  narrow leaf plantain (*Plantago lanceolata* L.,  Plantaginaceae) | Extraction of a fine powder of plant leaves with 70% ethanol, overnight, isolation of the extracted fraction by a muslin cloth, filtration, solvent rotary evaporation | Phenolics: cafeic acid,  protoctecuic acid, ferulic acid, sinapic acid, O-cumaric acid, flavonoid glycosides: rutin, myricetin, quercetin, kaempferol | - The most potent cytotoxic activity against CAL51 cell line with an IC_50_ of 23.7 μg/mL (for tested concentrations: 15, 31.25, 62.5, 125, 250, 500, 1000, 2000, 4000 μg/mL); - No noticeable cytotoxic effect of the extract against MDA-MB cells with a high IC_50_ of 250 μg/mL; - Inhibition of colony formation at extract high concentration – 4000 μg/mL only of CAL51 (selective suppression of the CAL51 proliferation), decrease in cells number treated with the extract due to cell detachment; - Condensed nuclei in the cells treated with extract indicating their early apoptosis (as compared to untreated cells) | (Alsaraf et al., 2019) |
| *Pancratium maritimum* L., Amaryllidaceae | Extraction (3 ×) of the fresh, crushed flowers with stalks in 70% ethanol, sonication, room temp., 6 h, filtration, combination of extracts, solvent rotary evaporation | Alkaloid –  pancratistatin | - Potent and selective growth inhibitory effect of pancratistatin against MDA-MB-231 with the IC_50_ of 0.14±0.00 μM | (Youssef et al., 2022) |
| Shrub (a medicinal plant) *Elaeagnus angustifolia* L.,  Elaeagnaceae | Extraction of the dried flowers by  boiling in autoclaved distilled water at 100 °C for 15 min, continuous  stirring, filtration | Phenolic acids and flavonoids; vitamins; proteins; elements: calcium, magnesium, potassium, and iron | - Significant inhibition of the proliferation of MDA-MB-231 and MDA-MB-436 cells in a dose-dependent manner (25, 50, 75, 100, 150, 200 μL/mL) for 48 h; for MDA-MB-231 cells inhibition by 30% and 55% for 100 and 200 μL/mL, respectively, for MDA-MB-436 cells, by 40 and 50%, respectively; - For 100 and 200 μL/mL: treated cells were characterized by loss of membrane integrity, deformation, cell shrinkage, contact inhibition, formation of apoptotic bodies; inhibition of cell-cycle progression; a significant reduction in the number of colonies for both cell lines | (Fouzat et al., 2022) |
| Meadow fleabane (*Inula britannica* L., Asteraceae) | Extraction of dried flowers with 95% ethanol, 12 h at reflux, partitioning of extract with petroleum ether, dichloromethane, and ethyl acetate | Sesquiterpenoids | - Antiproliferative activity of sesquiterpenoid (1,10β-dihydroxy-4αH-1,10-secoeudesma-5(6),11(13)-dien-12,8β-olide) against MDA-MB-231 cells with the IC_50_ of 11.5±0.7 μM, and MDA-MB-468 cells with the IC_50_ of 4.92±0.65 μM and of japonicone H for MDA-MB-468 cells with the IC_50_ of 6.68±0.70 μM after 48 h (tested doses: 0.1, 0.5, 1, 5, 10, 20 μM) | (Qi et al., 2022) |
| Dandelion (*Taraxacum mongolicum* Hand.-Mazz., *Taraxacum formosanum* Kitam., Asteraceae) | Preparation of aqueous extracts  and dissolution in boiled double distilled water | Phenolics: chicoric acid, flavonoids;  terpens: triterpenes,  sesquiterpenes;  polysaccharides; cyclic organic esters –lactones | - Cytotoxic effect of extract against MDA-MB-231 cells; higher cytotoxicity effect of *T. mongolicum* extract - Decrease in cell migration with the increase in extract dose (1, 2, 3 mg/mL) and colony formation for both extracts for 1 mg/mL, completely blocked colony formation at 2 mg/mL; - Reduction of cell proliferation, apoptosis induction, disruption of the mitochondrial membrane potential, and/or reduction of the oxygen consumption rate | (Lin et al., 2022) |
| Sea daffodil (*Pancratium maritimum* L., Amaryllidaceae) | Extraction of powdered plants from the flowering stage with methanol; extract suspension in 2% HCl, removal of the neutral material with diethylether, pH adjustment of the aqueous phase to 5 and successive extraction with dichloromethane, ethyl acetate, *n*-butanol | Amaryllidaceae alkaloids – e.g., lycorine, haemanthidine, haemanthamine;  fatty acid amides – alkamide; phenolics | - The IC_50_ of lycorine against MDA-MB-231 and MDA-MB-468 cells of 0.92 and 1.42 μM; the IC_50_ of haemanthidine towards MDA-MB-231 cells was 4.88 μM and 3.5 μM towards MDA-MB-468; for haemanthamine, these values were 3.95 μM and 3.8 μM, respectively; for 2α-10bα-dihydroxy-9-*O*-demethylhomolycorine, IC_50_ of 8.02 μM for MDA-MB-231 cells; - Treatment of MDA-MB-231 cells with 2α-10bα-dihydroxy-9-*O*-demethylhomolycorine resulted in induction of cells death by apoptosis, cell cycle arrest; increase in mitochondrial ROS generation | (Sancha et al., 2022) |

| **Type of natural product** | **Production method** | **Potential active metabolites** | ***In vitro* effect of extract on triple negative breast cancer cell lines** | **Ref.** |
| --- | --- | --- | --- | --- |
| **Tree/shrubs** | | | | |
| A shrub or small tree – *Prosopis juliflora* (Sw.) DC., Fabaceae | Soxhlet extraction of washed with tap and distilled water leaves, shade dried and powdered, in methanol, 8 h, 60 °C, solvent rotary evaporation | Phthalic acid and its esters | - Reduction in the % of cell survival in a time (up to 72 h) and dose-dependent (12.5, 25, 50, 100 μg/mL) manner, with the IC_50_ against MDA-MB-231 cells of 16.8 μg/mL; for this extract concentration, the following were observed: - morphological changes in MDA-MB-231 cells occurred: reduction in cell number and volume, shrinkage and appearance of floating or dead cells, rounding off; - significant inhibition in the migration of the MDA-MB-231 cells (71%) as compared to the control (DMSO^8^, 100%), as well as inhibition of the colony forming and the ability to regenerate; - excessive production of ROS in the cytoplasm of MDA-MB-231 cells treated with 16.8 μg/mL of extract up to 72 h; - MDA-MB-231 cells showed typical features of apoptosis with condensed chromatin and pyknotic nuclei (shrunken and dark) visible | (Utage et al., 2018) |
| A shrub or small tree –*Parkinsonia praecox* (Ruiz & Pav.) Hawkins, Fabaceae | Extraction of dried stems, berries and flowers separately with methanol (1:10 w/v), 4 days, occasional stirring, filtration, solvent rotary evaporation | Phenolics: quercetin, *p*-coumaric acid, flavonoids, tannins; terpenes; sugars | - Antiproliferative activity of *P. praecox* steam extract against MDA-MB-231 cells with the IC_50_ of 147±9 μg/mL; for berry and flower extract the IC_50_ > 400 μg/mL; - Steam extract contained the highest amount of phenolics , and the highest antioxidant activity (DPPH, ABTS^9^) | (López-Romero et al., 2022) |
| Aromatic shrub – *Lippia origanoides* Kunth,  Verbenaceae | Supercritical CO_2_ extraction of finely ground leaves and stems, next extraction of the material deposited on the chamber walls with methanol, sonication,15 min | Not indicated | - A dose-dependent (0.09, 0.11, 0.13, 0.15 mg/mL) cytotoxicity – a significant decrease in MDA-MB-231 cells viability at 0.09 mg/mL at 24 h; a dose of 0.12 mg/mL reduced cells viability to 51% at 24 h; at 72 h, a dose of 0.15 mg/mL reduced cells viability to < 2%; - In MDA-MB-231 cells, an *L. origanodes* extract caused fast and irreversible apoptosis | (Raman et al., 2018) |
| Tree – *Ficus crocata* (Miq.) Mart. ex Miq., Moraceae | Extraction (3 ×) of dried and ground leaves by maceration with  hexane (H), dichloromethane (D), acetone (A), 24 h, filtration, solvent rotary evaporation | (H): terpenes lup-20(29)-en-3-ol acetate and lupeol; (D): lupeol, the fatty acid 10,13,13-trimethyl-11-tetradecen-1-ol acetate, lup-20 (29)-en-3-ol  acetate; (A): β-sitosterol, stigmastan-3,5-dien | - Decrease in the number of cells at a concentration (5, 10, 20, 40, 80 μg/mL)- and time (24 and 48 h)- dependent manner – for 48 h, decrease in the number of cells for all tested concentrations, for 24 h only for 40, and 80 μg/mL; - All extracts decreased the proliferation of MDA-MB-231 cells with the strongest effect for (D) at a dose > 5 μg/mL (for (A) > 10 μg/mL, and for (H) > 20 μg/mL); - Antiproliferative activity: for (D) IC_50_ of 32.4 μg/mL, for (A) IC_50_ of 78.5 μg/mL, for (H) IC_50_ of 164 μg/mL at 48 h; - Induction of morphologic changes in MDA-MB-231 cells after use of (D) and (A) extracts (decrease in cell size, a rounded shape, the appearance of intracellular vacuoles that may indicate apoptosis) | (Sánchez-Valdeolívar et al., 2020) |
| Tree – *Eucalyptus nitens* (H.Deane & Maiden) Maiden, Myrtaceae | Extraction of the grounded outer bark of tree with dichloromethane | Triterpenic acids: betulinic acid, betulonic acid, oleanolic acid, ursolic acid; fatty acids; sterols  aliphatic alcohols | - Decrease in the viability of MDA-MB-231 cells exposed to *E. nitens* lipophilic extract in a dose (5, 10, 15, 20, 25 μg/mL) and time (24, 48 and 72 h)- dependent manner; at 48 h, the IC_50_ of 11.4±3.0 μg/mL and at 72 h, the IC_50_ of 6.29±1.30 μg/mL; - Incubation of cells with the extract resulted in the enhancement of the NAD+/NADH ratio likely due to a switch to mitochondrial respiration, which appears to be driven by amino acids and fatty acids produced by neutral lipid hydrolysis (triglycerides and cholesteryl esters) | (Guerra et al., 2021) |
| Common olive (*Olea europaea* L., Oleaceae) | Suspension of dried and pulverized leaves in water, microwave-assisted extraction (800 W, 10 min), filtration, solvent rotary evaporation | Oleuropein aglycone | - Antiproliferative and pro-apoptotic activity of oleuropein aglycone against MDA-MB-231 and tamoxifen-resistant MCF-7 (MCF-7/TAM) cells; - Significant inhibition of cell viability of both cell lines for deses 50 and 100 μM – IC_50_ of 70 μM for MCF-7/TAM and 53 μM for MDA-MB-231 cell line; - Induction of a cell cycle arrest in the G0/G1 phase, a reduction in the fraction of cells in S-phase, increase in the percentage of apoptotic cells | (Mazzei et al., 2020) |

| **Type of natural product** | **Production method** | **Potential active metabolites** | ***In vitro* effect of extract on triple negative breast cancer cell lines** | **Ref.** |
| --- | --- | --- | --- | --- |
| **Fungi** | | | | |
| Marine-derived fungus *Aspergillus unguis* (Émile-Weill & L. Gaudin) Thom & Raper 1934 (isolated from marine sponge) | Cultivation of fungus and sequential extraction of fungal cells with methanol and then dichloromethane, combination of both extracts | Depsidones – aspergillusidone D  and unguinol | - The inhibition of cell viability for unguinol with IC_50_ of 81 μM and was significantly higher than for aspergillusidone D with IC_50_ of 49 μM; - Statistically significantly reduction in the cells number in the S-phase and increase in the number of cells in the G2/M-phase after cells exposure to 100 μM of unguinol; - At a concentration of 60 μM, aspergillusidone D had no statistically significant impact on the MDA-MB-231 cells cycle status; - The depsidones have the ability to induce apoptosis | (Zwartsen et al., 2019) |
| Soil fungus – *Aspergillus niger*,  Aspergillaceae | Cultivation of fungus and ultrasound extraction of the mycelia with methanol, solvent rotary evaporation | Pyoluteorin  (a bichlorinated pyrrole) | - Significantly selective cytotoxicity of pyoluteorin against MDA-MB-231 cells with IC_50_ of 0.97±0.01 μM; for MDA-MB-468, the IC_50_ of 3.89±0.08 μM; - Arrest of the MDA-MB-231 cell cycle in the G2/M phase by pyoluteorin and induction of cell apoptosis (due to reduction in mitochondrial membrane potential, change in the expressions of apoptosis-related protein and ROS accumulation) | (Ding et al., 2020) |
| Edible fungus –*Gomphus purpuraceus* (Iwade) K. Yokoy. 1989,  Gomphaceae | Extraction of the dried and  pulverized mature fruit bodies of fungus with 95% ethanol | Alliacane sesquiterpenes –purpuracolide B  and purpuracolide C | - Both tested metabolites showed no inhibitory effect against the MDA-MB-231 cells with the IC_50_ > 20 μg/mL – they are inactive | (He et al., 2022) |
| Huaier (*Trametes robiniophila* Murrill 1907) –mushroom,  Polyporaceae | An aqueous extract (production method not indicated) | Not indicated | - Inhibition of the proliferation of tamoxifen-resistant cells (M7-TR) and fulvestrant-resistant cells (M7-FR); - A sharp decrease in cell viability at 8 mg/mL, independent of the treatment time (24, 48, 72 h); - A significant accumulation of both endocrine-resistant breast cancer cells in the G0/G1 phase (G0/G1 cell cycle arrest) | (Gao et al., 2017) |
| Lichen (*Physconia hokkaidensis* Kashiw. 1975,  Physciaceae) | Extraction of the dried lichen  thalli with methanol, room temp., 48 h using sonication, filtration, solvent rotary evaporation | Not indicated | - A dose-dependent (1, 3, 10, 30 μg/mL) cytotoxicity of extract against MDA-MB-231 cells with the IC_50_ of 23.8±1.2 μg/mL; - Reduction in the viability of MDA-MB-231 cells by cell cycle arrest at the sub-G1 phase due to the action of extract; - Induction of apoptosis of MDA-MB-231 cells | (Noh et al., 2021) |
| Lichen (*Parmotrema rampoddense* (Nyl.) Hale 1974,  Parmeliaceae) | Extraction of the air-dried, powdered lichen with methanol, room temp., filtration, solvent rotary evaporation | Atranorin (a depside lichen secondary metabolite) | - A dose-dependent (1–50 μM) cytotoxicity of atranorin against MDA-MB-231 cells with the IC_50_ of 5.36±0.85 μM at 48 h | (Harikrishnan et al., 2021) |
| Taiwan fungus –  *Antrodia cinnamomea* T.T. Chang & W.N. Chou 1995,  Polyporaceae | Extraction of powdered fruiting bodies with ethanol, soaking for 3 days, filtration, extraction of the  residue twice, combination of filtrates, solvent rotary evaporation | Triterpenoids – antcin K, antcin C, antcin B, methyl antcinate B, eburicoic acid, dehydroeburicoic acid | - Inhibition of the growth of breast cancer cells: MCF-7 cell and tamoxifen-resistant MCF-7 cell lines in a time (24, 48, 72 h)- and dose (100, 150, 200 μg/mL)-dependent manner with IC_50_ of 185 μg/mL and 196 μg/mL in 48 h, respectively; - A combination of extract with tamoxifen inhibited the proliferation of tamoxifen-resistant MCF-7 cells in a dose- and time-dependent manner, better than AC alone (tamoxifen cannot inhibit the proliferation of this cell line); - Significant induction of apoptosis in both breast cancer cell lines | (Lin et al., 2018) |

| **Type of natural product** | **Production method** | **Potential active metabolites** | ***In vitro* effect of extract on breast cancer cell lines** | **Ref.** |
| --- | --- | --- | --- | --- |
| **Algae/Seaweeds** | | | | |
| Red seaweed  (*Halymenia*  *durvillei* Bory de Saint-Vincent,  Halymeniaceae) | Extraction of the dried whole alga by maceration in 95% ethanol for 7 days, partitioning of the ethanolic extracts in hexane, solvent rotary evaporation | *n*-hexadecanoic acid, 6,10,14-trimethyl-2-pentadecanone, 9,12-octadecadienoic acid, heptadecane, hexadecenoic acid ethyl ester, oleic acid,  octadecanoic acid | - Cytotoxicity of extract against MDA-MB-231 cells with the IC_50_ of 50.4±17.7 μg/mL (for tested concentrations of 10–1000 μg/mL) for 24 h; - Algal extract can induce apoptotic MDA-MB-231 cells death (induction of mitochondrial damage) | (Sangpairoj et al., 2023) |
| Green seaweeds (*Ulva fasciata* FMPRC-100-5, *Ulva lactuca* FMPRC-100-6, Ulvaceae) | Immersion of the powdered seaweeds in methanol (70:30 v/v), room temp., darkness, for one week, continuous stirring, centrifugation, filtration, solvent rotary evaporation | Phenolics: flavonoid; Antioxidant activity - DPPH and FRAP^10^ assay | - The IC_50_ of the *U. fasciata* and *U. lactuca* extracts for the MDA-MB-231 cells was higher than 1000 μg/mL after 48 h of incubation and 804 and 849 μg/mL, respectively after 72 h; - Higher total phenolics and flavonoids content and antioxidant activity of *U. fasciata* extract than *U. lactuca* extract; - Significant reduction in the MDA-MB-231 cells viability for the *U. fasciata* – by 14.3% and 27.5% for 500 μg/mL, by 29.9% and 48.5% for 750 μg/mL and by 44.0% and 72.6% for 1000 μg/mL after 48 h and 72 h of incubation, respectively; - For *U. lactuca*, a significant reduction in the cell’s viability by 10.8%, 23.1% and 35.0% respectively, for 500, 750 and 1000 μg/mL after 48 h of incubation; and after 72 h of incubation these values were 20.3%, 44.0% and 68.3%, respectively | (Moulazadeh et al., 2021) |
| Brown seaweed (*Laminaria japonica* Areschoug, 1851, Laminariaceae) | Fucoidan – commercially available | Polysaccharide – fucoidan | - Modest inhibitory activity of fucoidan (0.125, 0.25, 0.5, 1, 2 mg/mL) against the viability of MDA-MB-231 cells; - 8% in MDA-MB-231 cells death after incubation for 48 h with 2 mg/mL of fucoidan (fucoidan preferentially decreased cellular growth rather than causing cell death); - Significant suppression (68%) of migration and invasion (83%) in MDA-MB-231 cells | (Hsu et al., 2020) |
| A single-cell green alga –  *Chlamydomonas reinhardtii* (CC-124),  Chlamydomonadaceae | Suspension of cultivated alga in 80% ethanol, maceration with mortar and pestle, transfer to water  bath at 80 °C, 4 h, solvent rotary evaporation | Sulfated polysaccharides;  Antioxidant activity – DPPH and ABTS assay | - Antioxidant, free radical scavenging ability, antiproliferative activity of sulfated polysaccharides against MDA-MB-231 cells; - Inhibition of MDA-MB-231 cells proliferation with the IC_50_ of 172 μg/mL; - Concentration-dependent (50, 100, 200, 400, 500 μg/mL) decrease in the number of MDA-MB-231 cell colonies (potential of polysaccharides to inhibit the clonal expansion of the cancer cells); - Cells treated with polysaccharide (400 μg/mL) underwent apoptosis | (Kamble et al., 2018) |

Abbreviations: ^1^DPPH, 2,2-diphenyl-1-picrylhydrazyl;  ^2^IC_50_, half-maximal inhibitory concentration; ^3^kDa, kilodaltons; ^4^TNBC, triple negative breast cancer; ^5^POLD1, DNA polymerase delta 1; ^6^LDH, lactate dehydrogenase; ^7^ROS, reactive oxygen species; ^8^DMSO, dimethyl sulfoxide; ^9^ABTS, 2,2′-azino-bis(3-ethylbenzothiazoline-6-sulfonic acid) diammonium salt; ^10^FRAP, ferric reducing antioxidant power assay

**Table S2.** Examples of *in vivo* investigations with natural products in triple negative breast cancer and HER-2 negative hormone-pretreated breast cancer models:

| **Type of natural product** | **Production method of a feed additive** | **Animals injected with**  **BC**^1^ **cells / treatment** | **Effects (as compared to the control group)** | **Ref.** |
| --- | --- | --- | --- | --- |
| Tree – *Prosopis juliflora* (Sw.), DC,  Fabaceae | Washed with tap and distilled water leaves were shade dried and powdered, Soxhlet extraction in methanol, 8 h, 60 °C, solvent rotary evaporation | - Normal (immune competent) female BALB/c mice (Mouse 4T1 breast tumor model), 6–8-week-old/20 mg/kg/day, 17 days | - Significant suppression of the 4T1-induced tumor growth by the extract; treatment slight reduction in the weight of the treated mice; - Treatment significantly decreased the size and weight of the 4T1-induced tumors; - Treated with extract tumors had cell shrinkage, nuclear pyknosis and intratumor spaces owing to death of cells | (Utage et al., 2018) |
| Moringa (*Moringa oleifera* Lam.,  Moringaceae) | Extraction of ground moringa seeds with water, 1:3 ratio, 2 h, 37 °C, addition of ethanol, filtration, solvent rotary evaporation | - Female mice with diet-induced obesity (MDA-MB-231 breast cancer xenograft model), 4-week-old / 0.6% w/w, 4 weeks | - Supplementation of moringa alone did not attenuate tumor growth as compared to chemotherapy alone; - Combination of moringa with chemotherapy worsened tumor progression; - Decrease in angiogenesis by moringa supplementation alone, but this effect was eliminated when chemotherapy was added | (Zunica et al., 2021) |
| Muscadine grape (*Vitis rotundifolia* Michx.,  Vitaceae) | An aqueous extract was prepared from powdered seeds and skin | - Female athymic mice, 6-week-old injected with actively growing human MDA-MB-231 cells / 0.1 mg of total phenolics/mL in drinking water (a dose of 0.5 mg total phenolics/25 g mouse /day), 4 weeks | - Polyphenol-rich extract reduced the proliferative markers Ki67 and cyclin D1 along with the tumor volume; - Extract reduced levels of cyclin D1 (through AKT^2^ and MAPK^3^ signaling pathways); - In MDA-MB-231 cells there was association between cyclin D1 reduction and cell cycle arrest | (Collard et al., 2020) |
| A perennial herb – *Prunella vulgaris* L. (PV), Lamiaceae | Extraction (3 ×) of the powdered material with boiling water, 1 h each (m/v, 1/10), filtration, solvent rotary evaporation  PV20, PV50 and PV95 – 20%, 50% and 95% ethanol eluted fraction | Female BALB/c mice (Mouse 4T1 breast tumor model), 5-week-old /PV20, PV50, PV95 – all fractions at 300 mg/kg, 21 days | - The tumor volume and weight in the experimental groups (PV20, PV50, PV95) was smaller than in the control group, especially in PV50; - In the PV50 and PV95 groups, necrotic cells were seen, and the tumor cells were loosely organized after treatment; - Increase in the number of vacuoles and decrease in the number of tumor cells in PV50 and PV95 groups as compared with the control group; - Cleaved Caspase-3 increased expression and nuclear DNA injury leading to apoptosis – induced by PV50 | (Luo et al., 2022) |
| An herbal medicine –*Polygonatum sibiricum* Redouté, Liliaceae | Soaking of the biomass overnight in water, heating at 95–100 ℃ with reflux for 1 h, filtration, procedure repeated 2 ×, combination of extracts, solvent rotary evaporation, addition of ethanol, maceration overnight, centrifugation, drying precipitate | Female BALB/c mice (Mouse 4T1 breast tumor model), 6–8 week-old / a  dose of 300 mg/kg day (100 μL every day), from day 11 to day 26 | - Significant reduction of the % of tumor-associated macrophages, tumor-infiltrating myeloid-derived suppressor cell (MDSC^4^) subpopulations, PMN-MDSC^5^ (CD11b^+^Ly6G^hi^) and M-MDSC^6^ (CD11b^+^Ly6C^hi^) in tumor microenvironment; - Hematopoietic cell expansion (caused by TNBC) in the spleen was inhibited; - Previously suppressed HSPCs and common lymphoid progenitors’ cells increased | (Xie et al., 2021) |
| Resveratrol  obtained from numerous species | Commercially available, production method not stated | Female BALB/c nude mice, 4–6 week-old and weighing between 18-22g. Logarithmically growing MDA-MB-231 and MDA-MB-231/POLD-OE cells (1 × 10^7^ cells/ml) inoculated subcutaneously.  8 resveratrol doses of a 25 mg/kg RSV solution. | - Resveratrol inhibited TNBC tumor growth in nude mouse models;. - POLD1^7^ overexpression attenuated the inhibitory effect; - Final tumor volume reduced, with notable necrosis in resveratrol-treated group;. - Immunohistochemistry showed reduced Proliferating Cell Nuclear Antigen and Ki-67, increased Cleaved-Caspase3; - POLD1 overexpression countered these effects. | (Liang et al., 2021) |

Abbreviations: ^1^BC, breast cancer; ^2^AKT, protein kinase B; ^3^MAPK, mitogen-activated protein kinase; ^4^MDSC, myeloid-derived suppressor cell; ^5^PMN-MDSC, neutrophils and polymorphonucler myeloid-derived suppressor cells; ^6^M-MDSCs, monocytic myeloid-derived suppressor cells; ^7^POLD1, DNA polymerase delta 1

Supplemental references

Alsaraf, K. M., Mohammad, M. H., Al-Shammari, A. M., and Abbas, I. S. (2019). Selective cytotoxic effect of Plantago lanceolata L. against breast cancer cells. *J Egypt Natl Canc Inst* 31, 1–7. doi: 10.1186/S43046-019-0010-3/FIGURES/4

Cao, X., Li, Y., Wang, Y., Yu, T., Zhu, C., Zhang, X., et al. (2022). Curcumin suppresses tumorigenesis by ferroptosis in breast cancer. *PLoS One* 17, e0261370. doi: 10.1371/JOURNAL.PONE.0261370

Cheng, X., Tan, S., Duan, F., Yuan, Q., Li, Q., and Deng, G. (2019). Icariin induces apoptosis by suppressing autophagy in tamoxifen-resistant breast cancer cell line MCF-7/TAM. *Breast Cancer* 26, 766–775. doi: 10.1007/S12282-019-00980-5

Collard, M., Gallagher, P. E., and Tallant, E. A. (2020). A Polyphenol-Rich Extract From Muscadine Grapes Inhibits Triple-Negative Breast Tumor Growth. *Integr Cancer Ther* 19, 1534735420917444. doi: 10.1177/1534735420917444

Cumaoglu, A., Bekci, H., Ozturk, E., Yerer, M. B., Baldemir, A., and Bishayee, A. (2018). Goji Berry Fruit Extracts Suppress Proliferation of Triple-Negative Breast Cancer Cells by Inhibiting EGFR-Mediated ERK/MAPK and PI3K/Akt Signaling Pathways. *Nat Prod Commun* 13, 701–706. doi: 10.1177/1934578X1801300613

Ding, T., Yang, L. J., Zhang, W. D., and Shen, Y. H. (2020). Pyoluteorin induces cell cycle arrest and apoptosis in human triple-negative breast cancer cells MDA-MB-231. *Journal of Pharmacy and Pharmacology* 72, 969–978. doi: 10.1111/JPHP.13262

Fouzat, A., Hussein, O. J., Gupta, I., Al-Farsi, H. F., Khalil, A., and Al Moustafa, A. E. (2022). Elaeagnus angustifolia Plant Extract Induces Apoptosis via P53 and Signal Transducer and Activator of Transcription 3 Signaling Pathways in Triple-Negative Breast Cancer Cells. *Front Nutr* 9, 418. doi: 10.3389/FNUT.2022.871667/BIBTEX

Gao, S., Li, X., Ding, X., Jiang, L., and Yang, Q. (2017). Huaier extract restrains the proliferative potential of endocrine-resistant breast cancer cells through increased ATM by suppressing miR-203. *Sci Rep* 7, 7313. doi: 10.1038/S41598-017-07550-9

Guerra, Â. R., Soares, B. I. G., Freire, C. S. R., Silvestre, A. J. D., Duarte, M. F., and Duarte, I. F. (2021). Metabolic Effects of a Eucalyptus Bark Lipophilic Extract on Triple Negative Breast Cancer and Nontumor Breast Epithelial Cells. *J Proteome Res* 20, 565–575. doi: 10.1021/ACS.JPROTEOME.0C00559

Harikrishnan, A., Veena, V., Lakshmi, B., Shanmugavalli, R., Theres, S., Prashantha, C. N., et al. (2021). Atranorin, an antimicrobial metabolite from lichen Parmotrema rampoddense exhibited in vitro anti-breast cancer activity through interaction with Akt activity. *J Biomol Struct Dyn* 39, 1248–1258. doi: 10.1080/07391102.2020.1734482

He, Y., Tan, A., Cai, X., Qin, Y., and Zhangshuang, D. (2022). Chemical constituents of Gomphus purpuraceus and its antiproliferative activities against cancer cells. *Mycosystema* 41, 991–998.

Ho, Y., Suphrom, N., Daowtak, K., Potup, P., Thongsri, Y., and Usuwanthim, K. (2020). Anticancer Effect of Citrus hystrix DC. Leaf Extract and Its Bioactive Constituents Citronellol and, Citronellal on the Triple Negative Breast Cancer MDA-MB-231 Cell Line. *Pharmaceuticals* 13, 1–17. doi: 10.3390/PH13120476

Hsu, W. J., Lin, M. H., Kuo, T. C., Chou, C. M., Mi, F. L., Cheng, C. H., et al. (2020). Fucoidan from Laminaria japonica exerts antitumor effects on angiogenesis and micrometastasis in triple-negative breast cancer cells. *Int J Biol Macromol* 149, 600–608. doi: 10.1016/J.IJBIOMAC.2020.01.256

Jaglanian, A., and Tsiani, E. (2020). Rosemary Extract Inhibits Proliferation, Survival, Akt, and mTOR Signaling in Triple-Negative Breast Cancer Cells. *Int J Mol Sci* 21, 810. doi: 10.3390/IJMS21030810

Kalebar, V. U., Hoskeri, J. H., Hiremath, S. V., and Hiremath, M. B. (2020). In vitro antiproliferative effect of aqueous extract of Solanum macranthum fruits on MDA-MB-231 tripple negative breast cancer cell line. *J Appl Biol Biotechnol* 8, 28–32. doi: 10.7324/JABB.2020.80105

Kamble, P., Cheriyamundath, S., Lopus, M., and Sirisha, V. L. (2018). Chemical characteristics, antioxidant and anticancer potential of sulfated polysaccharides from Chlamydomonas reinhardtii. *J Appl Phycol* 30, 1641–1653. doi: 10.1007/S10811-018-1397-2

Kombiyil, S., and Sivasithamparam, N. D. (2023). In Vitro Anti-cancer Effect of Crataegus oxyacantha Berry Extract on Hormone Receptor Positive and Triple Negative Breast Cancers via Regulation of Canonical Wnt Signaling Pathway. *Appl Biochem Biotechnol* 195, 2687–2708. doi: 10.1007/S12010-021-03724-4

Lee, M. M. L., Chan, B. D., Wong, W. Y., Qu, Z., Chan, M. S., Leung, T. W., et al. (2020). Anti-cancer Activity of Centipeda minima Extract in Triple Negative Breast Cancer via Inhibition of AKT, NF-κB, and STAT3 Signaling Pathways. *Front Oncol* 10, 491. doi: 10.3389/FONC.2020.00491

Liang, Z. J., Wan, Y., Zhu, D. D., Wang, M. X., Jiang, H. M., Huang, D. L., et al. (2021). Resveratrol Mediates the Apoptosis of Triple Negative Breast Cancer Cells by Reducing POLD1 Expression. *Front Oncol* 11, 569295. doi: 10.3389/FONC.2021.569295/BIBTEX

Lin, C. J., Chen, J. T., Yeh, L. J., Yang, R. C., Huang, S. M., and Chen, T. W. (2022). Characteristics of the Cytotoxicity of Taraxacum mongolicum and Taraxacum formosanum in Human Breast Cancer Cells. *Int J Mol Sci* 23, 11918. doi: 10.3390/IJMS231911918

Lin, Y. S., Lin, Y. Y., Yang, Y. H., Lin, C. L., Kuan, F. C., Lu, C. N., et al. (2018). Antrodia cinnamomea extract inhibits the proliferation of tamoxifen-resistant breast cancer cells through apoptosis and skp2/microRNAs pathway. *BMC Complement Altern Med* 18, 152. doi: 10.1186/S12906-018-2204-Y

Liu, H. C., Chiang, C. C., Lin, C. H., Chen, C. S., Wei, C. W., Lin, S. Y., et al. (2020). Anti-cancer therapeutic benefit of red guava extracts as a potential therapy in combination with doxorubicin or targeted therapy for triple-negative breast cancer cells. *Int J Med Sci* 17, 1015–1022. doi: 10.7150/IJMS.40131

López-Romero, J. C., Torres-Moreno, H., Valencia-Rivera, D. E., Leyva-Peralta, M. A., Lugo-Sepúlveda, R. E., Robles-Zepeda, R. E., et al. (2022). Nueva información del perfil de compuestos bioactivos, potencial antioxidante y antiproliferativo de Parkinsonia praecox (Fabaceae). *Acta Bot Mex* 128, e2089. doi: 10.21829/ABM129.2022.2089

Luo, H., Zhao, L., Li, Y., Xia, B., Lin, Y., Xie, J., et al. (2022). An in vivo and in vitro assessment of the anti-breast cancer activity of crude extract and fractions from Prunella vulgaris L. *Heliyon* 8, e11183. doi: 10.1016/J.HELIYON.2022.E11183

Maungchanburi, S., Rattanaburee, T., Sukpondma, Y., Tedasen, A., Tipmanee, V., and Graidist, P. (2021). Anticancer activity of Piper cubeba L. extract on triple negative breast cancer MDA-MB-231. *J Pharm Pharmacogn Res* 10, 39–51. doi: 10.56499/jppres21.1160_10.1.39

Mazzei, R., Piacentini, E., Nardi, M., Poerio, T., Bazzarelli, F., Procopio, A., et al. (2020). Production of Plant-Derived Oleuropein Aglycone by a Combined Membrane Process and Evaluation of Its Breast Anticancer Properties. *Front Bioeng Biotechnol* 8, 908. doi: 10.3389/FBIOE.2020.00908/BIBTEX

Mendonca, P., Darwish, A. G., Tsolova, V., El-Sharkawy, I., and Soliman, K. F. A. (2019). The Anticancer and Antioxidant Effects of Muscadine Grape Extracts on Racially Different Triple-negative Breast Cancer Cells. *Anticancer Res* 39, 4043–4053. doi: 10.21873/ANTICANRES.13560

Moulazadeh, A. A., Ranjbar, R., Hekmat, M., Sedaghat, F., Yousefzadi, M., and Najafipour, S. (2021). Comparison the cytotoxic effects of Ulva fasciata and Ulva lactuca on the MCF-7 and MDA-MB-231 breast cancer cell lines. *Physiology and Pharmacology* 25, 373–383. doi: 10.52547/phypha.25.4.2

Nguyen, C., Baskaran, K., Pupulin, A., Ruvinov, I., Zaitoon, O., Grewal, S., et al. (2019). Hibiscus flower extract selectively induces apoptosis in breast cancer cells and positively interacts with common chemotherapeutics. *BMC Complement Altern Med* 19, 98. doi: 10.1186/S12906-019-2505-9

Noh, J. I., Mun, S. K., Lim, E. H., Kim, H., Chang, D. J., Hur, J. S., et al. (2021). Induction of Apoptosis in MDA-MB-231 Cells Treated with the Methanol Extract of Lichen Physconia hokkaidensis. *J Fungi (Basel)* 7, 1–10. doi: 10.3390/JOF7030188

Qi, R. Y., Guo, C., Peng, X. N., and Tang, J. J. (2022). Sesquiterpenoids from Inula britannica and Their Potential Effects against Triple-Negative Breast Cancer Cells. *Molecules* 27, 5230. doi: 10.3390/MOLECULES27165230/S1

Raman, V., Aryal, U. K., Hedrick, V., Ferreira, R. M., Fuentes Lorenzo, J. L., Stashenko, E. E., et al. (2018). Proteomic Analysis Reveals That an Extract of the Plant Lippia origanoides Suppresses Mitochondrial Metabolism in Triple-Negative Breast Cancer Cells. *J Proteome Res* 17, 3370–3383. doi: 10.1021/ACS.JPROTEOME.8B00255

Sancha, S. A. R., Gomes, A. V., Loureiro, J. B., Saraiva, L., and Ferreira, M. J. U. (2022). Amaryllidaceae-Type Alkaloids from Pancratium maritimum: Apoptosis-Inducing Effect and Cell Cycle Arrest on Triple-Negative Breast Cancer Cells. *Molecules* 27, 5759. doi: 10.3390/MOLECULES27185759/S1

Sánchez-Valdeolívar, C. A., Alvarez-Fitz, P., Zacapala-Gómez, A. E., Acevedo-Quiroz, M., Cayetano-Salazar, L., Olea-Flores, M., et al. (2020). Phytochemical profile and antiproliferative effect of Ficus crocata extracts on triple-negative breast cancer cells. *BMC Complement Med Ther* 20, 191. doi: 10.1186/S12906-020-02993-6

Sangpairoj, K., Settacomkul, R., Siangcham, T., Meemon, K., Niamnont, N., Sornkaew, N., et al. (2023). Hexadecanoic acid-enriched extract of *Halymenia durvillei* induces apoptotic and autophagic death of human triple-negative breast cancer cells by upregulating ER stress. *Asian Pac J Trop Biomed* 12, 132. doi: 10.4103/2221-1691.338922

Utage, B. G., Shivajirao, P. M., Vasudeo, N. P., Shankar, K. S., and Nivarti, G. R. (2018). Prosopis juliflora (Sw.), DC induces apoptosis and cell cycle arrest in triple negative breast cancer cells: in vitro and in vivo investigations. *Oncotarget* 9, 30304–30323. doi: 10.18632/ONCOTARGET.25717

Xie, Y., Jiang, Z., Yang, R., Ye, Y., Pei, L., Xiong, S., et al. (2021). Polysaccharide-rich extract from Polygonatum sibiricum protects hematopoiesis in bone marrow suppressed by triple negative breast cancer. *Biomed Pharmacother* 137, 111338. doi: 10.1016/J.BIOPHA.2021.111338

Youssef, D. T. A., Shaala, L. A., and Altyar, A. E. (2022). Cytotoxic Phenylpropanoid Derivatives and Alkaloids from the Flowers of Pancratium maritimum L. *Plants* 11, 476. doi: 10.3390/PLANTS11040476/S1

Zunica, E. R. M., Yang, S., Coulter, A., White, C., Kirwan, J. P., and Gilmore, L. A. (2021). Moringa Oleifera Seed Extract Concomitantly Supplemented with Chemotherapy Worsens Tumor Progression in Mice with Triple Negative Breast Cancer and Obesity. *Nutrients* 13, 2923. doi: 10.3390/NU13092923

Zwartsen, A., Chottanapund, S., Kittakoop, P., Navasumrit, P., Ruchirawat, M., Van Duursen, M. B. M., et al. (2019). Evaluation of anti-tumour properties of two depsidones - Unguinol and Aspergillusidone D - in triple-negative MDA-MB-231 breast tumour cells. *Toxicol Rep* 6, 1216–1222. doi: 10.1016/J.TOXREP.2019.10.012
